# Non-coding RNA polymerases that silence transposable elements and reprogram gene expression in plants

**DOI:** 10.1080/21541264.2020.1825906

**Published:** 2020-11-12

**Authors:** Bart Rymen, Laura Ferrafiat, Todd Blevins

**Affiliations:** Institut de biologie moléculaire des plantes, Université de Strasbourg, Strasbourg, France

**Keywords:** RNA polymerase IV (Pol IV), non-coding RNA, RNA-directed DNA Methylation, transposable elements, plant gene regulation

## Abstract

Multisubunit RNA polymerase (Pol) complexes are the core machinery for gene expression in eukaryotes. The enzymes Pol I, Pol II and Pol III transcribe distinct subsets of nuclear genes. This family of nuclear RNA polymerases expanded in terrestrial plants by the duplication of Pol II subunit genes. Two Pol II-related enzymes, Pol IV and Pol V, are highly specialized in the production of regulatory, non-coding RNAs. Pol IV and Pol V are the central players of RNA-directed DNA methylation (RdDM), an RNA interference pathway that represses transposable elements (TEs) and selected genes. Genetic and biochemical analyses of Pol IV/V subunits are now revealing how these enzymes evolved from ancestral Pol II to sustain non-coding RNA biogenesis in silent chromatin. Intriguingly, Pol IV-RdDM regulates genes that influence flowering time, reproductive development, stress responses and plant–pathogen interactions. Pol IV target genes vary among closely related taxa, indicating that these regulatory circuits are often species-specific. Data from crops like maize, rice, tomato and *Brassica* *rapa* suggest that dynamic repositioning of TEs, accompanied by Pol IV targeting to TE-proximal genes, leads to the reprogramming of plant gene expression over short evolutionary timescales.

## Transposable element silencing: DNA methylation meets RNA interference

Early DNA association studies revealed that eukaryotic genomes are full of repetitive sequences, hinting that most chromosomal DNA does not code for proteins [1,2]. Advances in molecular genetics and DNA sequencing revealed that most non-coding DNA consists of transposable elements (TEs), mobile genetic parasites that excise or copy themselves to then insert elsewhere in the genome. TEs represent 40% of the human genome, 20% of the *Arabidopsis thaliana* genome and ~80% of crop genomes such as *Zea mays* (maize), *Hordeum vulgare* (barley) and *Triticum aestivum* (wheat) [3–6]. In the course of evolution, TEs can generate useful genetic diversity [7,8], but on shorter timescales TE insertions cause deleterious mutations and genomic instability [9,10].

Animal and plant cells express elaborate molecular surveillance systems to recognize and silence TEs. A common mechanism of TE silencing involves dimethylation of histone H3 at lysine 9 (H3K9me2) along with methylation of cytosines in DNA, a chromatin state typically refractory to RNA polymerase II (Pol II) transcription [5]. Non-coding RNAs guide such repressive chromatin marks to specific TE targets. In metazoans, chromatin-level TE surveillance is driven by ~26–32 nt Piwi-interacting RNAs (piRNAs), though many animal taxa do not methylate their DNA [11]. In plants, the enzymatic machinery for piRNAs is absent, but an analogous pathway mediated by ~24 nt small interfering RNAs (siRNAs) triggers RNA-directed DNA methylation (RdDM). RdDM is a functionally specialized, nuclear RNA interference pathway that evolved in terrestrial plants.

Plants express enzymes that methylate cytosines in three sequence contexts referred to as CG, CHG, and CHH sites (where H is A, C, or T). The enzyme METHYLTRANSFERASE 1 (MET1) is a maintenance methyltransferase that copies CG methylation from parent to daughter strands during plant DNA replication [12], like its mammalian ortholog, DNMT1. The plant CHROMOMETHYLASES, CMT2 and CMT3, maintain DNA methylation by “reading” histone methylation marks and catalyzing CHH or CHG methylation in adjacent DNA [13–15]. Finally, *de novo* methylation is mediated by either DOMAINS REARRANGED METHYLTRANSFERASE (DRM; CG, CHG and CHH sites) or DNA METHYLTRANSFERASE 3 enzymes in plants (DNMT3; CG and CHH sites). In the bryophyte *Physcomitrella patens*, PpDNMT3b is the major *de novo* methyltransferase at CG and CHH sites, with PpDRMs playing only a minor role [16]. By contrast, DRM activity is crucial for *de novo* methylation in flowering plants, because DNMT3 is absent in these species [16–18]. *A. thaliana* has two known *DRM* genes, *DRM1* and *DRM2*; the double mutant *drm1 drm2* abolishes RNA-directed DNA methylation [19]. The higher expression of *DRM2* compared to *DRM1*, and the fact that *drm2* single mutants recapitulate the late-flowering phenotype of *drm1 drm2* double mutants, suggest that DRM2 is the key *de novo* methyltransferase in *A. thaliana* [20].

Four pioneering studies in 2005 reported the discovery of Pol IV and Pol V as a key specialized transcription machinery for RdDM [21–24]. Pol IV and Pol V are enzymes that assemble from unique combinations of Pol II-like subunits that evolved ~470 million years ago in the terrestrial plant lineage [25–28]. Pol IV and Pol V transcription activities converge to ensure that DRM2 methylates appropriate targets. Pol II mostly transcribes genes in pursuit of mRNA biogenesis. By contrast, Pol IV and Pol V transcribe TE loci, intergenic repeats and the promoter regions of certain genes. The consensus in the field is that Pol IV synthesizes precursors for siRNAs that guide RdDM [29,30], whereas Pol V transcribes loci into non-coding scaffold RNAs that are critical for target recognition [31].

In this review, we first describe how Pol IV and Pol V orchestrate TE surveillance, which is a central function of RdDM in plants. Then, we survey what is known about Pol IV-specific subunits, their internal domain structure, unique protein partners and emerging findings about what brings the Pol IV pathway together in the nucleus. Finally, we present a survey of novel biological functions of Pol IV-RdDM that have been discovered in recent years.

### *Pol IV and Pol V non-coding transcripts guide* de novo *DNA methylation*

**Figure 1.**
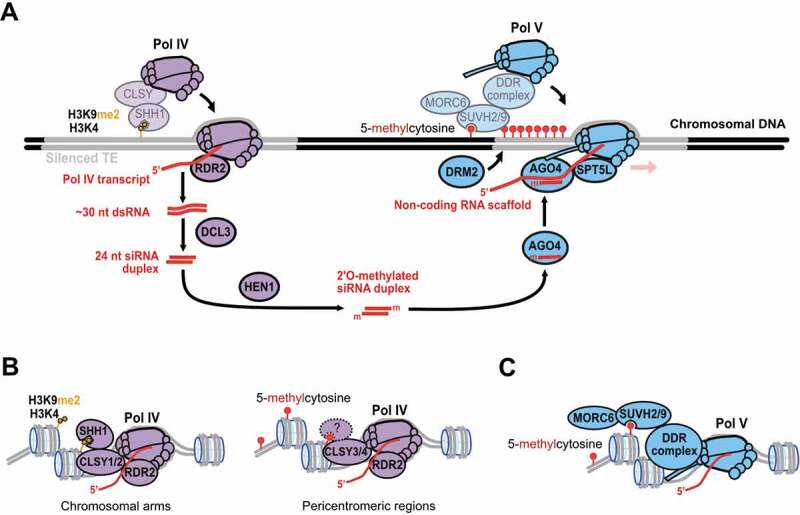
**Schematic overview of the RNA-directed DNA methylation (RdDM) pathway**. (a) Pol IV transcripts are processed by RDR2 into double-stranded RNA (dsRNA), which is diced into a 24 nt small interfering RNA (siRNA) duplex by DCL3. HEN1 performs 2'O-methylation of each siRNA strand. AGO4-siRNA complexes bind to complementary sequences in nascent Pol V transcripts, and this AGO4-siRNA-Pol V complex is stabilized by the interaction of the NRPE1 (Pol V) CTD with AGO4, and of Pol V with SPT5L. Finally, this leads to the recruitment of DRM2, which catalyzes *de novo* cytosine methylation. (b) The Pol IV-RdDM pathway is initiated by recruitment of Pol IV to silent chromatin; this typically occurs in distal chromosomal regions by the dimethylated Histone 3 Lysine 9 (H3K9me2) reader, SHH1, which interacts with Pol IV through chromatin remodelers CLSY1 or CLSY2. In pericentromeric regions, CLSY3 and CLSY4 are required for Pol IV recruitment, which may interact with these DNA regions using a DNA methylation reader, so far unknown in a direct or indirect fashion. (c) Pol V is recruited to chromosomal targets by a dedicated machinery, mostly different from the factors required for Pol IV transcription. SUVH2 and SUVH9 are SET and RING-associated (SRA) domain proteins thought to recruit Pol V to regions of methylated DNA. The DDR complex (DRD1, DMS3 and RDM1; not detailed here) serves as a bridge complex that mediates Pol V transcription at many, if not all RdDM targets. Pol V interactions with the target DNA and chromatin are further consolidated by MORC6

A combination of genetic and biochemical experiments has shown that Pol IV non-coding RNA transcripts initiate siRNA biogenesis for RdDM (Figure 1a) [29,32–35]. The Pol IV complex is physically coupled to the enzyme RNA-DEPENDENT RNA POLYMERASE 2 (RDR2), one of six different RDRs expressed in *A. thaliana*. The Pol IV-RDR2 partnership is one key difference between the RdDM pathway and other functionally distinct small RNA pathways [30,35,36]. This protein–protein interaction enables channeling of Pol IV primary transcripts to RDR2 for double-stranded RNA (dsRNA) synthesis *in vivo* and *in vitro* (Figure 1a) [29,33,35].

The Pol IV primary transcripts and RDR2’s dsRNA products are not detectable by northern blotting of RNA from wild-type plants, nor are they seen in conventional RNA-seq, which makes them challenging to detect [29,32]. This suggests that RDR2 products are very efficiently processed into 24 nt siRNAs by DICER-LIKE 3 (DCL3). However, Pol IV-RDR2 products accumulate *in vivo* when Dicer processing is disrupted in *dcl3* single mutant or in *dcl2 dcl3 dcl4* triple mutant plants [29,32,34]. The RDR2 products are relatively short (~26–45 nt) both *in vivo* and *in vitro*, and have a 3'overhang of 1–2 non-templated nucleotides, attributable to RDR2’s terminal transferase activity [29,33]. These properties of Pol IV-RDR2 products have the logical consequence that DCL3 can dice each such dsRNA substrate only once to generate a single 24 nt siRNA duplex [29,32].

The short length of Pol IV-dependent RNAs is likely due to their unusual termination mechanism. Pol IV transcript termination does not rely upon specific signal sequences akin to other RNA polymerases. Pol IV termination could, instead, be primarily determined by the geometry of its transcription bubble, because Pol IV is ineffective at displacing non-template DNA in transcription assays. According to this model, Pol IV transcribes single-stranded DNA in a conventional bubble (~18–25 bases for eukaryotic Pol II [37]), but then encounters base-paired DNA at the bubble’s edge and only extends a further ~12–18 nt before terminating to release ~30–43 nt transcripts, as was observed *in vitro* [33], in close agreement with *in vivo* RNA-seq data [29].

After RDR2 synthesizes dsRNAs from Pol IV primary transcripts, DCL3 dices these dsRNAs into 24 nt siRNAs, the enzyme HEN1 catalyzes 2'O-methylation at siRNA 3' ends, and the siRNAs are loaded onto ARGONAUTE4 (AGO4) (Figure 1a). In plants, the gene families encoding RNA interference factors have diversified: four to five genes encode DCL proteins and over 10 genes encode AGO proteins. The mechanism governing the specific function of DCL3 in processing dsRNA products of RDR2 is not clear, but a preference of DCL3 to dice short, 30–50 nt dsRNAs with a 5'-terminal adenine has been reported from *in vitro* assays [38]. The mature 24 nt siRNAs are primarily loaded onto AGO4 [39] and to lesser extents onto AGO6 and AGO9 [40–42]. The specificity of Pol IV-RDR2 derived 24 nt siRNAs for AGO4 is not fully understood, but the affinity of AGO4 for a 5'-terminal adenine corresponds to features of Pol IV-RDR2 derived siRNAs [43].

During the RdDM effector phase, siRNA sequence-specific DNA methylation depends on Pol V transcription of the target locus (Figure 1a) [31]. Pol V transcribes chromosomal loci into long non-coding RNAs that more closely match the DNA template sequence than Pol IV transcripts [29,44–46]. Nascent Pol V transcripts are scaffolds to which AGO4-siRNA complexes physically associate; logically, this could occur via base-pairing of the AGO4-loaded siRNA guide to complementary transcripts [47]. In addition, the Pol V largest subunit (NRPE1) possesses a carboxy-terminal domain (CTD) that interacts with AGO4 [48]; the Pol V partner protein, SPT5L, also interacts with AGO4 [49]. The NRPE1 CTD and SPT5L, combined, provide independent functions that consolidate AGO4-Pol V association [50,51]. Formation of the AGO4-Pol V-SPT5L complex is thought to attract DRM2, which catalyzes DNA methylation [17]. How cycles of AGO4-siRNA-transcript tethering are coupled to DRM2 methylation is unclear, but it could involve co-transcriptional cleavage of Pol V transcripts by AGO4’s slicer activity [44,52]. For a subset of targets, DRM2 methylation also depends on AGO4 interaction with three RNA binding proteins (IDN2, IDNL1 and IDNL2; not depicted in Figure 1) [53–55].

The partnership between Pol IV and Pol V steps creates an RdDM positive feedback loop [21,23] by amplifying the silent chromatin marks required for the recruitment of each RNA polymerase [56,57]. At most targets, the synthesis of high levels of 24 nt siRNAs depends on this full RdDM cycle, including the Pol V-AGO4-DRM2 effector step [23,52,58,59]. The importance of Pol IV-Pol V cooperation was directly tested by the artificial targeting of Pol IV and Pol V to the same locus [60]. Because Pol IV and Pol V are both needed for robust RdDM, a potential consequence of this cooperative mechanism is the prevention of ectopic silencing linked to DNA methylation spreading. Pol V transcription is prominent at the edges of RdDM targets, such as TE boundaries, which limits the action of the RdDM pathway while repressing Pol II transcription [45,61]. Together, these findings all illustrate the importance of recruiting Pol IV and Pol V to appropriate genomic loci.

Several factors have been identified that may recruit Pol IV to chromosomal targets (Figure 1b). Mass spectrometry found that CLASSY proteins, a subfamily of SWI2/SNF2-like ATPases related to chromatin remodelers, copurify with the Pol IV complex in *A. thaliana* and maize [36,62]. The biochemical activity of CLSY proteins has not been elucidated, but genetic screens have isolated *clsy* mutations that disrupt gene silencing in both these plant species [63–65]. The four *A. thaliana* proteins, CLSY1 through CLSY4, facilitate the association of Pol IV at about 90% of loci that give rise to 24 nt siRNAs [66]. Intriguingly, CLSY1 and CLSY2 mainly facilitate Pol IV association at distal loci in the *A. thaliana* chromosome arms, while CLSY3 and CLSY4 assure this function in dense pericentromeric heterochromatin [66].

CLSY1 and CLSY2 may provide protein–protein interactions that bridge Pol IV to its key partner protein, SAWADEE HOMEODOMAIN HOMOLOG 1 (SHH1) [36,66]. SHH1 would read repressive H3K9me2 marks at targets via its SAWADEE domain, recruiting CLSY1/2 and Pol IV to silent chromatin (Figure 1b, left-hand diagram) [56,67]. Supporting this model, SHH1’s SAWADEE domain selectively interacts with H3K9me2 and unmethylated H3K4 on peptide arrays, the 24 nt siRNA clusters requiring SHH1 overlap with those requiring CLSY1/2, and Pol IV complex copurification with SHH1 depends on CLSY1/2 [56,66]. By contrast, biogenesis of 24 nt clusters at CLSY3 and CLSY4-dependent loci does not correlate with a reduction in H3K9me2 in mutants implicated in H3K9 methylation. Mutants defective in CG methylation do cause a loss of CLSY3 and CLSY4-dependent 24 nt clusters, though, suggesting that a DNA methylation reader is directly or indirectly involved [66]. No epigenetic readers for CLSY3/4-dependent guidance of Pol IV to pericentromeric regions have yet been identified (Figure 1b, right-hand diagram).

At the downstream effector step, two SU(VAR)3–9 homolog class proteins, SUVH2 and SUVH9, appear to recruit Pol V to genomic regions marked by DNA methylation (Figure 1c). SUVH2 and SUVH9 are histone methyltransferase-like proteins that have lost their intrinsic methyltransferase activity, but that can bind methylated DNA via a conserved SET and RING-ASSOCIATED (SRA) domain [57]. Furthermore, SUVH2 and SUVH9 interact with the microrchidia adenosine triphosphatase proteins MORC1 and MORC6 to assist Pol V recruitment to chromatin [68]. MORC6 mediates heterochromatin condensation at certain loci, thereby contributing to the silencing effects of RdDM independently of DNA methylation [69–71]. Interaction of SUVH2 and SUVH9 with Pol V occurs via the DDR complex [68].

The DDR complex consists of three proteins, DEFECTIVE IN RNA-DIRECTED DNA METHYLATION 1 (DRD1), DEFECTIVE IN MERISTEM SILENCING 3 (DMS3), and RNA-DIRECTED DNA METHYLATION 1 (RDM1), which are essential for Pol V recruitment and transcription *in vivo* [31,72–74]. The DDR complex core is an RDM1 dimer with plant-specific protein folds but enigmatic biochemical features [74–76]. This RDM1 dimer serves as a bridge to recruit two DMS3 dimers, which are proteins homologous to hinge domain regions of cohesin and condensin ATPases [77]. Finally, a putative SWI2/SNF2 chromatin remodeler, DRD1, is recruited resulting in an ordering of the coiled-coil helix of the DMS3 dimers [74,78]. The SWI2/SNF2 ATPase domain of DRD1 could plausibly allow it to interact with chromatin, but how the DDR complex gets recruited to Pol V or SUVH2/9 is unknown.

### More than a sum of Pol II parts: unique Pol IV and Pol V subunits

**Figure 2.**
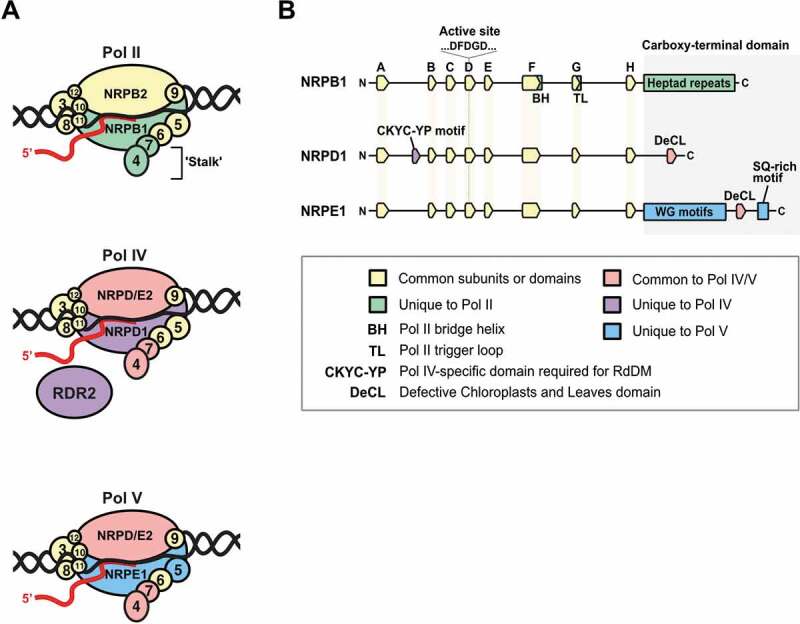
**Pol IV and Pol V evolved from Pol II but have specific subunits and domains**. (a) Pol II, Pol IV and Pol V are composed of 12 subunits (respectively called NRPB, NRPD and NRPE from 1 to 12). Certain subunits are common to all three complexes (yellow); others are unique to Pol II (green), Pol IV (purple) or Pol V (blue); and a few assemble with Pol IV and Pol V but not with Pol II (pink). The 4^th^ and 7^th^ subunits of nuclear RNA polymerases form a stalk domain. (b) NRPB1, NRPD1 and NRPE1, the largest subunits of Pol II, Pol IV and Pol V, contain several conserved domains (A to H). NRPB1 contains specific domains (green): the bridge helix, trigger loop (lost in NRPD1 and NRPE1) and heptad repeats in its carboxy-terminal domain (CTD). The DEFECTIVE CHLOROPLASTS AND LEAVES (DeCL) domain is common to both Pol IV and Pol V (red). NRPD1 contains a specific CKYC-YP motif between the A and the B domain (purple). The NRPE1 CTD contain WG motifs and a SQ-rich domain (blue)

Pol IV and Pol V have evolved from Pol II [25,28,35]. Consequently, these plant-specific enzymes are composed of 12 subunits, comparable to Pol II (Figure 2a). However, Pol IV and Pol V contain distinct catalytic subunits, interact with a unique set of recruitment factors, target mostly non-genic loci, and generate products with novel biological functions. Logically, these peculiarities of Pol IV and Pol V must be reflected in their protein structure. Phylogenetic and structure-function analyses have probed the composition of these specialized RNA polymerases to determine how the plant non-coding RNA transcription machinery governs RdDM and genome surveillance.

The architecture of eukaryotic RNA polymerases has been deeply investigated in yeast. Pol II was the first such enzyme solved at atomic resolution via x-ray crystallography [79–81]. High-resolution structures are now available for Pol I and Pol III as well [82–84]. Pol I, Pol II and Pol III have two core catalytic subunits, with a total of 14, 12 and 17 subunits, respectively [85]. Decades of intense study have identified structural elements that are conserved across these RNA polymerases, and often also in archaeal and bacterial RNA polymerases [86,87]. The RNA polymerase complex consists of a crab-claw shape, with clamp and the jaw structures allowing opening and closing of the primary channel [88,89]. Two highly conserved metal-binding sites (Metal A and Metal B) chelate Mg^2+^ ions necessary for DNA-templated base addition, which proceeds 5' to 3' using ribonucleotide triphosphates as substrates. Formation of the Metal A site requires three aspartates of the largest subunit (e.g., NRPB1 in Pol II) arranged in a conserved DFDGD motif (Figure 2b) [79,90]. Other conserved structural elements include the fork loop (s), rudder, wall, trigger loop and bridge helix allow the basic mechanism of transcription. In addition, all polymerase have a protruding “stalk” structure, composed of two peripheral subunits, which promotes the formation of an open complex and increases processivity [86,91].

Pol IV and Pol V subunit composition resembles Pol II: each enzyme is composed of 12 subunits, about half of which are encoded by the same genes (Figure 2a) [25]. The plant RNA polymerase subunits are named NUCLEAR RNA POLYMERASE x (NRPx1 to NRPx12) proteins. In this nomenclature, the RNA polymerase complex is indicated by x’s position in the Latin alphabet (A for Pol I, B for Pol II, C for Pol III, D for Pol IV and E for Pol V). NRPB1 and NRPB2 are Pol II’s two largest subunits, together forming the catalytic core. Hence, NRPD1 and NRPD2 form the Pol IV core, and NRPE1 and NRPE2 form the Pol V core. In *A. thaliana*, the 2nd subunits of Pol IV and Pol V are encoded by a single gene *NRPD/E2*, whose gene product can assemble to form either Pol IV (NRPD1 + NRPD/E2) or Pol V (NRPE1 + NRPD/E2) (Figure 2a). Similarly, the 4th and 7th subunit heterodimer of the Pol IV stalk is distinct from the heterodimer associated with Pol II. Again, a particular subunit combination (NRPD/E4 + NRPD/E7) is inferred to form the stalk that is functional in either Pol IV or Pol V, but not in Pol II [25,62]. The 5th subunit of Pol V is specialized (NRPE5), whereas Pol IV competes with Pol II for the same 5th subunit (NRPB/D5). Finally, certain subunits are common to all these RNA polymerases: NRPB/D/E3, NRPB/D/E6, NRPB/D/E8, NRPB/D/E10, NRPB/D/E11 and NRPB/D/12 subunits are each encoded by common genes and can assemble with Pol II, Pol IV or Pol V (Figure 2a). Based on the many mutually orthologous subunits in Pol IV, Pol V and Pol II, it is hypothesized that the general structure and assembly of the core enzymes is evolutionary conserved [25,62]. The discovery of common assembly factors for Pol IV, Pol V and Pol II, called MINIYO (IYO) and QUATRE QUART 2 (QQT2) supports this hypothesis [92].

Despite being expressed from different genes, the largest subunits of Pol II (NRPB1), Pol IV (NRPD1) and Pol V (NRPE1) have similar primary structures (Figure 2b). In these largest subunits, the eight domains (A to H) that are conserved in Pol I, Pol II and Pol III [93,94] are also found in NRPD1 and NRPE1, including the aspartate triad (DFDGD motif) located in the D domain that forms the Metal A binding site essential for the catalytic activity of all RNA polymerases [23,35]. The difference between the largest subunits is mainly positioned at their carboxy-terminal domains (CTDs). The Pol II CTD, containing ~25 to 52 tandem copies of a conserved heptad peptide (34 repeats in *A. thaliana*), is known to be involved in Pol II recruitment and is subject to phosphorylation important for different transcriptional steps (activation, elongation, termination) [95,96].

By contrast, Pol V’s CTD is intrinsically disordered and contains varying numbers of glycine and tryptophan (WG/GW) motifs (17 repeats in *A. thaliana*), known as “AGO-hooks” that stabilize the interaction of AGO4-clade proteins with Pol V [48,97,98]. Additionally, the DEFECTIVE CHLOROPLASTS AND LEAVES (DeCL) domain in the NRPE1 CTD is important for Pol V transcription *in vivo* [99]. In Pol V, the DeCL domain and adjacent glutamine-serine (QS) repeats mediate binding of a 3'–>5' exoribonuclease, RRP6L1 [99]. The trimming action of RRP6L1 on Pol V transcripts could potentially lead to a pausing of Pol V in chromatin, necessary for robust RdDM [99,100]. A DeCL domain is also present in the CTD of the Pol IV subunit NRPD1. Pol IV mutants missing this DeCL domain display reduced transcription activity, resulting in an ~80% loss of Pol IV-dependent siRNA production and corresponding quantitative losses in RdDM across the entire genome [101]. The exact role of each DeCL domain in their distinct Pol IV and Pol V enzyme contexts will be an interesting avenue for investigation in coming years.

We recently discovered that Pol IV harbors a novel amino acid motif in its NRPD1 N-terminus, which is absent in Pol II but highly conserved in Pol IV [102]. This motif is composed of a C[KR]YC box followed by a 5–10 amino acid spacer, then by a YPx[MV][KR]F[KR] box (Figure 2b). A point mutation in the motif caused a loss in 24 nt siRNA biogenesis, disrupted *de novo* DNA methylation and reactivated TE loci in *A. thaliana* [102]. Beyond its critical role in genome surveillance and DNA methylation patterning, the precise function of the motif is not fully understood. Residual 24 nt siRNAs accumulate at TE extremities and other hotspots in the epigenomic landscape of the C[KR]YC-box mutant, suggesting that these could be sites of RdDM initiation. One attractive model is that the Pol IV-specific motif in NRPD1’s N-terminus governs the mechanism of silent chromatin amplification as RdDM spreads across a locus in WT plants [102].

While having gained novel protein motifs and domains, plant NRPD1 and NRPE1 subunits have also shed structures that are highly conserved in eukaryotic NRPB1. In the NRPB1 G domain, there is a structural element called the trigger loop (Figure 2b) [103] that is significantly modified in NRPD1 and NRPE1 [35,104]. The trigger loop is not essential for Pol II *in vitro* transcription activity, but its deletion from NRPB1 causes reduced transcription fidelity [105,106]. The absence in the NRPD1 G domain of otherwise conserved amino acids is a plausible explanation for the high error rate of Pol IV transcription [35,46]. The trigger loop is the direct target of the fungal toxin α-amanitin, a Pol II inhibitor, explaining Pol IV’s lack of sensitivity to this drug [35,105].

Substantial progress has been made over the last 15 years, since the discovery of Pol IV and Pol V.A concrete molecular understanding has emerged. These plant non-coding RNA polymerases have unique subunit combinations, functional domains, specialized motifs and other features that distinguish them from Pol II and from each other. Yet, the domains in Pol IV that mediate its assembly with the partner enzyme RDR2, or that assure Pol IV recruitment via SHH1 and CLSY remain unknown. The structures in Pol V that mediate its specific association with the DDR complex and SUVH2/SUVH9 also need to be identified. Furthermore, much remains to be discovered about the Pol IV and Pol V transcription cycles: their precise requirements for recruitment, transcription initiation, elongation and termination. In the future, a comprehensive structure-function analysis of unique domains in Pol IV and Pol V will be highly informative, especially in relation to specific protein–protein interactions that allow the assembly of unique subunit combinations with their specialized partners.

### Diverse biological functions of Pol IV and RNA-directed DNA methylation

**Figure 3.**
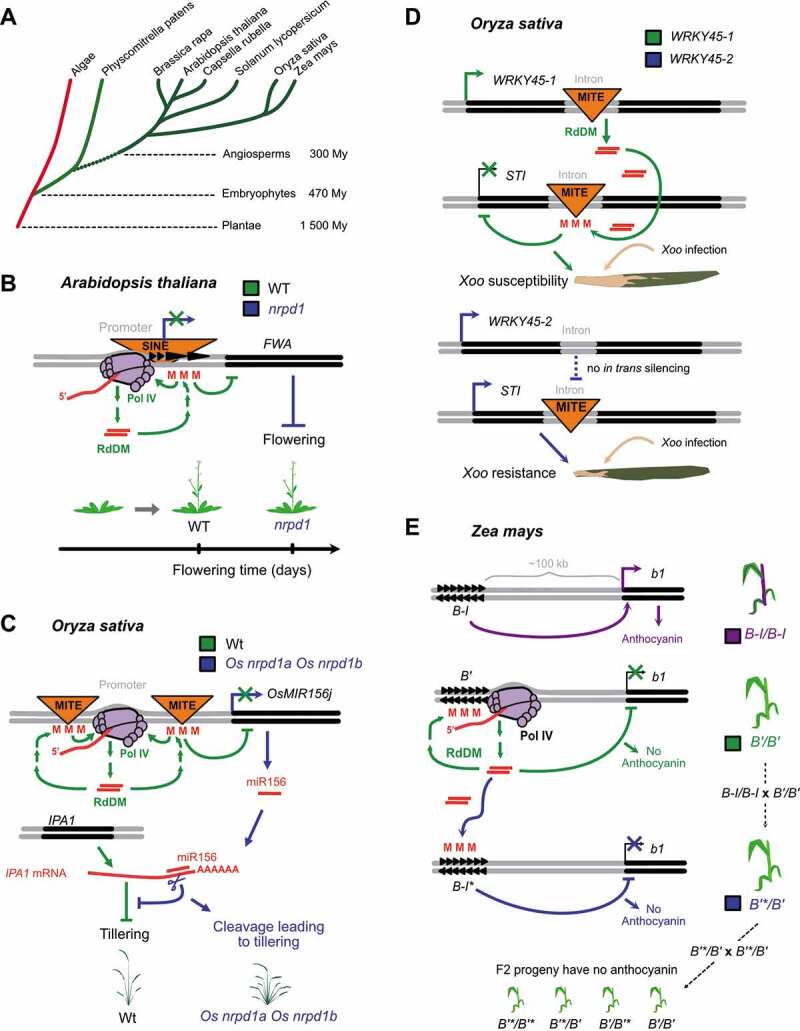
**The genome surveillance function of RNA polymerase IV (Pol IV) has been coopted for plant gene regulation**. (a) Evolutionary tree representing the plant species reported to have *de novo* DNA methylation by Pol IV and RNA-directed DNA Methylation (RdDM). Dashed lines indicate the predicted timescale of plant diversification in million years (My) [208]. Highlighted in green are the species in which genes encoding RdDM players are present. Red indicates that no evidence for genes encoding RdDM factors has been reported. (b) Tandem repeats similar to transposable elements (TEs) in the *FWA* gene promoter allow Pol IV and RdDM to repress *FWA* expression for flowering time regulation in the *Arabidopsis* genus. (c) Insertion of Miniature Inverted-repeat Transposable Elements (MITEs) near gene loci can regulate gene expression in *Oryza sativa* (rice). Pol IV represses a miRNA precursor gene, *OsMIR156j*, in wild-type rice. Transcriptional silencing of *OsMIR156j* is disrupted in *Os nrpd1a Os nrpd1b* mutant plants, causing miR156 to overaccumulate and target the mRNA of *Os* IPA1, which ultimately leads to increased tillering. (d) Plant siRNAs can also target and transcriptionally repress genes *in trans*, for example to regulate innate immunity in rice. Expression of the *Os STI* gene leads to *Xanthomonas oryzae* pv. *oryzae* (Xoo) resistance. In *Xoo* susceptible plants harboring the *WRKY45-1* allele, an intronic MITE triggers production of siRNAs via Pol IV-RdDM that will guide DNA methylation to a homologous MITE sequence in an intron of *STI* gene and silence it. In *Xoo* resistant plant harboring the *WRKY45-2* allele, the intronic MITE and resultant silencing of *STI* is missing. (e) In maize, paramutation depends on the Pol IV and RDR2 enzyme machinery for production of 24 nt siRNAs. The parental “paramutagenic” *B’* allele is linked to a silent *booster 1* (*b1*) locus, whereas *b1* is still expressed in the case of a *B-I* “paramutable” allele. When *B-I/B-I* (purple) and *B’/B’* (green) individuals are crossed to form the *B-I/B’* genotype in F1 plants, siRNAs from *B’* are thought to silence the *B-I* allele *in trans*, thereby changing *B-I* into silent B’* and shutting down anthocyanin production. The DNA methylation induced by *B’* is heritable to all F2 progeny (B’*/B’*, B’*/B’, B’/B’* or *B’/B’* genotypes), and newly formed B’* alleles are also paramutagenic in future crosses

Phylogenetic analyses have identified genes encoding Pol IV and Pol V subunits in species throughout the terrestrial plant lineage (Embryophyta), suggesting that Pol IV-RdDM evolved in a common ancestor of land plants (Figure 3a) [28,107–109]. Supporting this hypothesis, the analysis of Pol IV subunit mutations has revealed Pol IV-dependent siRNA biogenesis and *de novo* DNA methylation in the moss *Physcomitrella patens* [110] and in diverse angiosperms, including tomato (*Solanum lycopersicum*) [111], rice (*Oryza sativa*) [112–114], maize (*Zea mays)* [104,115–117], *Brassica rapa* [118] and *Capsella rubella* [119].

Although the mechanism of Pol IV function is conserved across diverse plant species, the biological consequences of RdDM defects are not. Deficiency phenotypes range from developmental defects in *pol IV* mutants of maize [120,121], tomato [111] and rice [112,113], to altered responses to heat, UV or drought stress [122–124], to susceptibility to various biotic challenges [125–129]. The most common defects affect flowering and sexual reproduction [130]. *pol IV* mutants in *B. rapa, C. rubella*, tomato and rice each showed decreased fertility [111,112,118,119]. Furthermore, maize *pol IV* mutants (*rmr6* [120]) are defective in sex determination, and *rdr2* mutants (*mop1* [121]) display floral defects.

Looking at three different species of the Brassicaceae family, RdDM deficiency causes reproductive defects of varying severity. In *pol IV* mutants of *C. rubella,* both female and male gametes show abnormalities consistent with the high rate of seed abortion [119], whereas only female reproductive cells are disrupted in *pol IV* mutants of *B. rapa* [118]. The few viable seeds in *pol IV* mutants of *C. rubella* and *B. rapa* are abnormally small. Despite this reduced seed size in *pol IV* mutants of *A. thaliana*, however, fertility is not significantly impaired [118,131]. The diversity of *pol IV* mutant phenotypes suggests that the genomic targets causing these defects may differ between species.

At least two molecular mechanisms could explain the pleiotropy of deficiency phenotypes in *pol IV* mutants. A first possibility is that disrupting Pol IV-RdDM derepresses silent chromatin leading to TE insertion mutations in developmental and stress regulatory genes. However, MET1-dependent maintenance methylation and associated heterochromatin are often sufficient to silence TEs under standard growth conditions. Outside of stress conditions and chemical treatments, TE mobilization is rarely observed in *pol IV* single mutants [132–136]. By contrast, novel TE insertions are frequent in plants lacking DNA methylation maintenance (e.g., *ddm1* or *met1* null mutants), and especially in plants defective in both RdDM and maintenance pathways [137–140]. The possibility exists, of course, that the balance between CG methylation maintenance and Pol IV-RdDM differs among plant species, explaining the severe defects caused by *pol IV* mutants in species other than *A. thaliana.*

A more likely explanation of why *pol IV* mutants affect reproduction and stress responses in species-specific ways is that RdDM targets gene promoter-proximal TEs. Because of the variation in TE distribution in plant genomes, *pol IV* mutations in different taxa could trigger pleiotropic phenotypes due to misexpression of different sets of genes [137–140]. Promoter-proximal TEs provoke various regulatory outcomes, but the most common is transcriptional silencing of a gene promoter because of RdDM at the adjacent TE (Figure 3b). In the *Arabidopsis* genus, for example, the *FLOWERING WAGENINGEN* (*FWA*) gene promoter contains direct repeats reminiscent of a SINE TE, which attracts the RdDM machinery [21,131,141,142,143,144,145]. *FWA* is a maternally imprinted gene that encodes a repressor of flowering [146]. Consequently, *FWA* gene activation in vegetative tissues of RdDM-deficient plants, such as *pol IV* null mutants, causes late flowering (Figure 3b).

Comparable mechanisms explain the phenotypic consequences of RdDM deficiency in rice (Figure 3c). In *pol IV* null mutants of rice (*Os nrpd1a Os nrpd1b*), loss of siRNAs from the miniature inverted-repeat TEs (MITEs) flanking a microRNA precursor gene is linked to miR156 overaccumulation [112]. Ectopic miR156 can then target the mRNA of IDEAL PLANT ARCHITECTURE 1 (IPA1), a key repressor of tillering [147]. Deregulation of RdDM affecting this miR156-*IPA1* developmental pathway is thus thought to cause increased tillering in *Os nrpd1a Os nrpd1b* mutants (Figure 3c). Similarly, because of the MITEs in genes important for phytohormone biosynthesis, gibberellin and brassinosteroid levels are perturbed in *Os dcl3* and *Os rdr2* knock-down lines, leading to stunted plant growth [113]. In maize, genome-wide association studies for drought tolerance revealed that RdDM targets a MITE insertion located in the *Zm NAC111* gene promoter [124]. The resulting silencing of *Zm NAC111* causes reduced drought tolerance in temperate maize. These studies show that RdDM can silence TE-proximal genes *in cis*, modifying economically important traits in cereals.

RdDM also acts *in trans*, as was documented using viroid, virus and transgene-induced systems in the 1990s [148–150], leading to the discovery of the Pol V effector machinery [22,77,78]. Such *in trans* RdDM is known to modify the expression of natural plant genes with consequences for disease resistance. In rice, MITE polymorphisms in an intron of the *WRKY45* gene correlate with rice susceptibility to the *Xanthomonas oryzae* pv. *oryzae* (*Xoo*) bacterial infection and attenuate resistance to *Magnaporthe oryzae* fungus (Figure 3d) [129]. Zhang and colleagues showed that MITE siRNAs derived from an intron in the *WRKY-1* allele associated with *Xoo* susceptibility can target a homologous MITE sequence in the unrelated *STI* gene (Figure 3d). Pol IV involvement was not directly tested, but DNA methylation of the *STI* locus requires Os RDR2 and Os DCL3 functions. RdDM suppression of *STI* expression leads to a crippled defense against *Xoo* infection in rice subspecies with the *WRKY45-1* locus harboring this MITE insertion.

In maize, Pol IV-dependent siRNAs can target genes in a process called paramutation [151]. Paramutation is an interaction between different alleles of the same locus that results in non-Mendelian inheritance: after a cross the silent “paramutagenic” allele triggers heritable silencing of the other, “paramutable” allele. This *in trans* effect results in the inheritance of two silent alleles without changing the DNA sequence of either [152]. A famous case is the *booster1* (*b1*) locus, which encodes a transcription factor that promotes anthocyanin biosynthesis. Maize plants expressing *b1* display purple coloration (Figure 3e). There is an enhancer consisting of seven tandem repeats situated ~100 kb upstream of *b1*, which likely forms a complex secondary structure to activate *b1*. This enhancer comes in two allelic forms: the active *B-I* paramutable allele (*B-I/B-I* genotypic plants are purple) and *B’*, a silent paramutagenic allele (*B’/B’* genotypic plants are green). Crossing *B-I/B-I* to *B’/B’* plants changes *B-I* into a heritable silent allele B’*. The B’*/B’ plants of the F1 generation are all green, because both copies of the *b1* locus are inactive. Strikingly, all subsequent F2 progeny (B’*/B’*, B’*/B’, B’/B’* and *B’/B’* genotypes) are also green, rather than including the ¼ purple plants expected for Mendelian inheritance of unmodified *B-I* (Figure 3e).

Pol IV transcription, RDR2 production of dsRNA and subsequent siRNA biogenesis seem to be critical for paramutation, because genetic lesions in the largest subunit of maize Pol IV (MOP3/RMR6) [104,153], in the second largest subunit of maize Pol IV and Pol V (MOP2/RMR7) [115,116], in maize RDR2 (MOP1) [117,121] or in maize CLSY (RMR1) [154] each disrupt *B-I* to B’* paramutation. The best model to explain these results is that Pol IV-dependent siRNAs from the *B’* paramutagenic allele target DNA methylation and silencing of this *B’* allele *in cis*, preventing *b1* locus activation (Figure 3e, green arrows). The same *B’*-derived siRNAs would induce *in trans* DNA methylation at the paramutable *B-I* allele in heterozygous plants (Figure 3e, blue arrows). This DNA methylation is heritable, with all F2 progeny of the B’*/*B’* heterozygote showing the same green phenotype as the original *B’*/*B’* parent and heterozygous F1 generation. Why RdDM does not continuously target the tandem-repeat enhancer natively present in *B-I*/*B-I* parent plants is unresolved. Pol IV-dependent paramutation has also been observed for other loci that regulate pigment biosynthesis in maize [155], with analogous phenomena occasionally reported in other plant species [156].

Together, these examples show that distinct genes are silenced by Pol IV-RdDM in different plant species. In analogy to miRNAs, Pol IV can modulate temporal and spatial patterns of gene expression, with essential functions in reproductive development. The regulation of flowering by *FWA* silencing occurs in multiple species [144]. More typically, however, RdDM targets do not show synteny or sequence similarity across species. Comparison of Pol IV-dependent siRNA clusters in *A. thaliana* to those inferred to exist in *A. lyrata* (based on 24 nt siRNA profiling) revealed only limited conservation [157]. More recently, comparison of gene expression in wild type and *pol IV* mutant plants from two Brassicaceae species (*A. thaliana* and *C. rubella*) found only negligible overlap in RdDM-targeted genes [119]. Unlike the many cases of ancient plant miRNA–target mRNA pairs, Pol IV-dependent siRNAs rarely target genes in an evolutionary conserved manner across species [158,159]. Instead, the dynamic reprogramming of gene expression via RdDM, observed under stress conditions [122,160] and during key phases in reproduction [161–164] may allow plants to evolve and adapt to challenging conditions in a stochastic manner over shorter timescales.

## Conclusions and future directions

**Figure 4.**
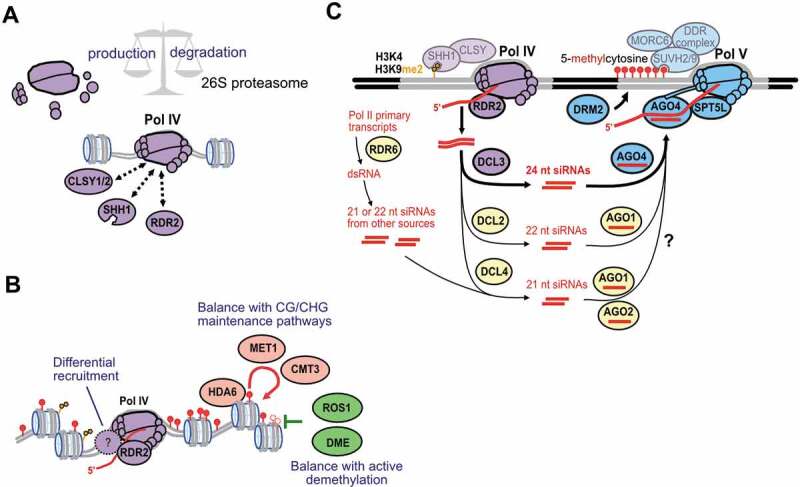
**Factors that regulate, initiate and counterbalance Pol IV function in RdDM**. (a) Components of the Pol IV-RdDM machinery could be regulated by transcriptional control of Pol IV subunit/partner genes, post-transcriptional silencing of the corresponding mRNAs, post-translational modification of the individual proteins, or targeted degradation of the Pol IV complex itself. Pol IV assembly and turnover is likely governed by the specific subunits and functional domains that mediate Pol IV’s interactions with SHH1, CLSYs, RDR2 and yet unknown, specialized regulatory proteins. (b) Initial recruitment of Pol IV to specific sites in the genome may require factors other than SHH1 and CLSY proteins, which still remain to be discovered. Another important process that controls the intensity of Pol IV-RdDM is the balance between CG/CHG methylation maintenance (involving MET1, CMT3, and HDA6 proteins), and active 5-methylcytosine removal by plant glycosylase lyases (ROS1 and DME). (c) In addition to the canonical RdDM pathway involving Pol IV-RDR2-DCL3 and 24 nt siRNAs loaded onto AGO4 (bold arrows), other pathways can trigger *de novo* DNA methylation in plants (thin arrows). The alternatives include Pol II-RDR6 production of dsRNAs that are diced into 21–22 nt siRNAs, or Pol IV-RDR2 production of dsRNAs that are diced by the alternate enzymes DCL2 and DCL4, into 22 and 21 nt siRNAs, which tend to associate with different effectors, such as AGO1 and AGO2, to guide DNA methylation

Pol IV and Pol V are plant-specific RNA polymerases that evolved from an ancestral Pol II into enzymes specialized in generating different non-coding RNAs for RdDM. The most evident biological function of Pol IV-RdDM is TE silencing (Figure 1). In addition, the balancing of paternal-maternal imprinting appears to be an evolutionary conserved function of RdDM, as reviewed elsewhere [165,166]. Plant species have also coopted RdDM, in different ways, to regulate the expression of TE-proximal genes in key biological pathways (Figure 3) [112,113,116,118–120]. Whether Pol IV and Pol V are regulated in a spatio-temporal manner to optimize distinct RdDM functions in TE silencing, imprinting, developmental control and stress response is an open question (Figure 4a).

Protein components of RdDM are not constitutively expressed in *A. thaliana*, showing higher expression in the shoot apical meristem cells [167] and during late embryogenesis [161]. In addition, abiotic and biotic cues from the environment influence the expression and stability of RdDM proteins. For instance, heat shock dramatically decreases the expression of several RdDM pathway components, including *NRPD1* and *NRPE1* [160]. Moreover, *Rice grassy stunt virus* hijacks the host plant’s ubiquitin-proteasome system to degrade Os NRPD1A (Pol IV) proteins and modify plant development (Figure 4a) [168]. Similarly, the cell cycle regulatory anaphase promoting factor (APC) can mediate the degradation of DMS3, a core component of the DDR complex needed for Pol V function [169]. However, the expression patterns of RdDM players do not necessarily correspond to locations of siRNA action, because siRNAs can act non-cell-autonomously; differential regulation of siRNA translocation could thus play a role in RdDM control [170–175]. Further tissue-specific investigations of RdDM will be needed to understand how Pol IV and Pol V transcription are coupled in time and space to ensure appropriate deposition of DNA methylation during development, or in response to environmental stress conditions.

In addition to global changes in the accumulation of RdDM players induced by stress or developmental cues, local spatio-temporal changes in the activity of RdDM components seem likely. For instance, during UV-C light-induced DNA damage, an abrupt increase in Pol IV-dependent siRNA levels is observed at damaged sites [123], suggesting that Pol IV is recruited to these sites upon damage. It is not yet known whether differential expression of Pol IV recruitment factors or local changes in chromatin status help explain Pol IV function at sites of DNA damage (Figure 4b). The local activity of RdDM is further reinforced or antagonized by alternative epigenetic pathways. Best understood in this respect are the DNA methylation maintenance pathways, which often target the same types of loci as those subject to RdDM (Figure 4b) [176]. In addition, histone deacetylation by HDA6 [177] and histone demethylation by JMJ14 [178,179] are critical for TE silencing and Pol IV function at subsets of RdDM targets.

Changes in partially redundant pathways might explain the variable importance of RdDM in different regulatory and chromatin contexts. For example, MET1, DDM1 and HDA6-dependent pathways counterbalance Pol IV to regulate the intensity of RdDM in ribosomal RNA gene tandem repeats [180–182]. Chromatin remodeling factors [183,184] have the potential to affect the recruitment of RdDM, while DNA demethylases [127,185–190] and Pol II transcriptional factors [135] are known to antagonize the RdDM silencing mechanism (Figure 4b). Future genetic analyses and artificial co-targeting strategies [60] will help dissect the complex interplay between the multiple layers of chromatin modification and non-coding RNA regulation in plants.

An alternative explanation for the variation in regulatory function of the RdDM pathway across plant species might lie in recent duplications and subfunctionalizations of RdDM protein families. Several of the Pol IV and Pol V subunits, as well as other proteins of the RdDM pathway, are encoded by a variable number of gene paralogs across species. Most notable are duplications of the largest and second largest Pol IV/V-like subunits in cereal monocots compared to eudicot plants [62,191]. Although the molecular and biological functions of these extra RNA polymerase subunits remain unknown, they might reinforce or even extend the RdDM machinery in species like rice, barley and maize.

In addition, molecular pathways have been recently described in *A. thaliana* that use varying combinations of the core Pol IV-RdDM components. For instance, Pol IV was found to mediate the biogenesis of 21 nt siRNAs at DNA double-strand breaks [192] or at photodamage-induced lesions [123]. Similar to Pol IV transcripts during RdDM, the siRNA precursors at DNA damage sites are RDR2-dependent, but the downstream processing step requires DCL4 rather than DCL3 [123,192]. Resulting 21 nt siRNAs are loaded onto AGO1 in the case of photodamage [123] or onto AGO2 in the case of DNA double-strand breaks (Figure 4c)[192]. These siRNA-AGO complexes could facilitate DNA damage recognition [123,192] and prevent excessive alterations of the DNA methylation landscape upon photodamage [193].

In fact, siRNAs derived from Pol II transcription also trigger DNA methylation via the Pol V effector machinery. RDR6, an enzyme paralogous to RDR2, uses Pol II transcripts from TEs or transgenes to synthesize dsRNAs that are diced into 21 and 22 nt siRNAs by DCL4 and DCL2, respectively, [138,194,195]. Genetic experiments suggest that these siRNAs guide AGO1 (or other AGOs) to sites of Pol V transcription for non-canonical RdDM (Figure 4c)[196]. Moreover, endogenous Pol II transcripts that fold into hairpin RNAs or miRNA precursors are at times processed by DCL2, DCL3 or DCL4, again leading to non-canonical RdDM [196–200]. Intriguingly, Pol II and Pol V can dynamically modify chromatin topology in response to the hormone auxin, via synthesis of *APOLO* long non-coding RNA in *A. thaliana* [201]. To what extent the abovementioned alternative Pol II/Pol IV/Pol V functions are evolutionarily conserved is unknown, but DNA repair proteins like DDB2 and silencing factors like RDR6 have orthologs throughout terrestrial plants [202–205]. The discovery of diverse non-canonical siRNA pathways thus opens exciting new avenues for exploring Pol IV and Pol V transcription in model and crop species.

Both the canonical Pol IV-RdDM pathway (Figure 4c, bold arrows) and emerging variant pathways (Figure 4c, light arrows) are likely to exploit specific subunit variants or structural changes in plant non-coding RNA polymerases. The largest subunits of Pol IV and Pol V, and their common stalk domains each possess unique amino acid sequences and highly divergent domains (Figure 2) [23,25,108,206,207]. Theoretically, these unique subunit features must combine to account for the distinctive biochemical activities of Pol IV, of its partners SHH1, CLSY and RDR2, as well as of the related Pol V complex (Figure 4c) [33,35]. As described above, researchers have begun exploring non-catalytic domains and isolating hypomorphic mutations in novel residues to test their contribution to the non-coding RNA specialization of Pol IV and Pol V [48,50,99,101,102]. Further structure-function analyses will be needed to fully understand the subunit assemblies of Pol IV and Pol V, and how their activities are regulated during plant growth and development. Finally, advances in protein purification and cryo-electron microscopy will, no doubt, one day reveal the precise structures of Pol IV and Pol V and give exquisite insights into their functional specialization.

## References

[1] Britten RJ, Kohne DE. Repeated sequences in DNA. Science. 1968;161:529–540. 4874239 10.1126/science.161.3841.529

[2] Ohno S. So much “junk” DNA in our genome. Brookhaven Symp Biol. 1972;23:366–370. 5065367

[3] Wicker T, Gundlach H, Spannagl M, et al. Impact of transposable elements on genome structure and evolution in bread wheat. Genome Biol. 2018;19. DOI:10.1186/s13059-018-1479-0. PMC6097303 30115100

[4] Wicker T, Schulman AH, Tanskanen J, et al. The repetitive landscape of the 5100 Mbp barley genome. Mob DNA. 2017;8:22. 29270235 10.1186/s13100-017-0102-3 PMC5738225

[5] Slotkin RK, Martienssen R. Transposable elements and the epigenetic regulation of the genome. Nat Rev Genet. 2007;8:272–285. 17363976 10.1038/nrg2072

[6] SanMiguel P, Tikhonov A, Jin YK, et al. Nested retrotransposons in the intergenic regions of the maize genome. Science. 1996;274:765–768. 8864112 10.1126/science.274.5288.765

[7] Kazazian HH. Mobile Elements: drivers of Genome Evolution. Science. 2004;303:1626–1632. 15016989 10.1126/science.1089670

[8] Lisch D. How important are transposons for plant evolution? Nat Rev Genet. 2013;14:49–61. 23247435 10.1038/nrg3374

[9] Ong-Abdullah M, Ordway JM, Jiang N, et al. Loss of karma transposon methylation underlies the mantled somaclonal variant of oil palm. Nature. 2015;525:533. 26352475 10.1038/nature15365 PMC4857894

[10] Belancio VP, Deininger PL, Roy-Engel AM. LINE dancing in the human genome: transposable elements and disease. Genome Med. 2009;1:97. 19863772 10.1186/gm97 PMC2784310

[11] Ozata DM, Gainetdinov I, Zoch A, et al. PIWI-interacting RNAs: small RNAs with big functions. Nat Rev Genet. 2019;20:89–108. 30446728 10.1038/s41576-018-0073-3

[12] Saze H, Scheid OM, Paszkowski J. Maintenance of CpG methylation is essential for epigenetic inheritance during plant gametogenesis. Nat Genet. 2003;34:65–69. 12669067 10.1038/ng1138

[13] Cao X, Aufsatz W, Zilberman D, et al. Role of the DRM and CMT3 methyltransferases in RNA-directed DNA methylation. Curr Biol. 2003;13:2212–2217. 14680640 10.1016/j.cub.2003.11.052

[14] Stroud H, Do T, Du J, et al. Non-CG methylation patterns shape the epigenetic landscape in Arabidopsis. Nat Struct Mol Biol. 2014;21:64–72. 24336224 10.1038/nsmb.2735 PMC4103798

[15] Zemach A, Kim MY, Hsieh P-H, et al. The Arabidopsis nucleosome remodeler DDM1 allows DNA methyltransferases to access H1-containing heterochromatin. Cell. 2013;153:193–205. 23540698 10.1016/j.cell.2013.02.033 PMC4035305

[16] Yaari R, Katz A, Domb K, et al. RdDM-independent de novo and heterochromatin DNA methylation by plant CMT and DNMT3 orthologs. Nat Commun. 2019;10:1613. 30962443 10.1038/s41467-019-09496-0 PMC6453930

[17] Zhong X, Du J, Hale CJ, et al. Molecular mechanism of action of plant DRM de Novo DNA methyltransferases. Cell. 2014;157:1050–1060. 24855943 10.1016/j.cell.2014.03.056 PMC4123750

[18] Zhang H, Lang Z, Zhu J-K. Dynamics and function of DNA methylation in plants. Nat Rev Mol Cell Biol. 2018;19:489–506. 29784956 10.1038/s41580-018-0016-z

[19] Stroud H, Greenberg MVC, Feng S, et al. Comprehensive analysis of silencing mutants reveals complex regulation of the Arabidopsis Methylome. Cell. 2013;152:352–364. 23313553 10.1016/j.cell.2012.10.054 PMC3597350

[20] Cao X, Jacobsen SE. Role of the Arabidopsis DRM methyltransferases in de Novo DNA Methylation and gene silencing. Curr Biol. 2002;12:1138–1144. 12121623 10.1016/s0960-9822(02)00925-9

[21] Pontier D. Reinforcement of silencing at transposons and highly repeated sequences requires the concerted action of two distinct RNA polymerases IV in Arabidopsis. Genes Dev. 2005;19:2030–2040. 16140984 10.1101/gad.348405 PMC1199573

[22] Kanno T, Huettel B, Mette MF, et al. Atypical RNA polymerase subunits required for RNA-directed DNA methylation. Nat Genet. 2005;37:761–765. 15924141 10.1038/ng1580

[23] Onodera Y, Haag JR, Ream T, et al. Plant nuclear RNA polymerase IV mediates siRNA and DNA methylation-dependent heterochromatin formation. Cell. 2005;120:613–622. 15766525 10.1016/j.cell.2005.02.007

[24] Herr AJ. RNA polymerase IV directs silencing of endogenous DNA. Science. 2005;308:118–120. 15692015 10.1126/science.1106910

[25] Ream TS, Haag JR, Wierzbicki AT, et al. Subunit compositions of the RNA-silencing enzymes Pol IV and Pol V reveal their origins as specialized forms of RNA polymerase II. Mol Cell. 2009;33:192–203. 19110459 10.1016/j.molcel.2008.12.015 PMC2946823

[26] Kaul S, Koo HL, Jenkins J, et al. Analysis of the genome sequence of the flowering plant Arabidopsis thaliana. Nature. 2000;408:796–815. 11130711 10.1038/35048692

[27] Pikaard CS, Haag JR, Ream T, et al. Roles of RNA polymerase IV in gene silencing. Trends Plant Sci. 2008;13:390–397. 18514566 10.1016/j.tplants.2008.04.008 PMC2679257

[28] Luo J, Hall BD. A multistep process gave rise to RNA polymerase IV of land plants. J Mol Evol. 2007. DOI:10.1007/s00239-006-0093-z 17160640

[29] Blevins T, Podicheti R, Mishra V, et al. Identification of pol IV and RDR2-dependent precursors of 24 nt siRNAs guiding de novo DNA methylation in arabidopsis. Elife. 2015;4:1–22. 10.7554/eLife.09591 PMC4716838 26430765

[30] Xie Z, Johansen LK, Gustafson AM, et al. Genetic and functional diversification of small RNA pathways in plants. PLoS Biol. 2004;2:e104. 15024409 10.1371/journal.pbio.0020104 PMC350667

[31] Wierzbicki AT, Haag JR, Pikaard CS. Noncoding transcription by RNA polymerase Pol IVb/Pol V mediates transcriptional silencing of overlapping and adjacent genes. Cell. 2008;135:635–648. 19013275 10.1016/j.cell.2008.09.035 PMC2602798

[32] Zhai J, Bischof S, Wang H, et al. A one precursor one siRNA model for Pol IV-dependent siRNA biogenesis. Cell. 2015;163:445–455. 26451488 10.1016/j.cell.2015.09.032 PMC5023148

[33] Singh J, Mishra V, Wang F, et al. Reaction mechanisms of Pol IV, RDR2, and DCL3 drive RNA channeling in the siRNA-directed DNA methylation pathway. Mol Cell. 2019;75:576–589.e5. 31398324 10.1016/j.molcel.2019.07.008 PMC6698059

[34] Li S, Vandivier LE, Tu B, et al. Detection of Pol IV/RDR2-dependent transcripts at the genomic scale in Arabidopsis reveals features and regulation of siRNA biogenesis. Genome Res. 2015;25:235–245. 25414514 10.1101/gr.182238.114 PMC4315297

[35] Haag JR, Ream TS, Marasco M, et al. In Vitro transcription activities of Pol IV, Pol V, and RDR2 reveal coupling of Pol IV and RDR2 for dsRNA synthesis in plant RNA silencing. Mol Cell. 2012;48:811–818. 23142082 10.1016/j.molcel.2012.09.027 PMC3532817

[36] Law JA, Vashisht AA, Wohlschlegel JA, et al. SHH1, a homeodomain protein required for DNA methylation, as well as RDR2, RDM4, and Chromatin remodeling factors, associate with RNA polymerase IV. PLoS Genet. 2011;7:e1002195. 21811420 10.1371/journal.pgen.1002195 PMC3141008

[37] Barnes CO, Calero M, Malik I, et al. Crystal structure of a transcribing RNA polymerase II complex reveals a complete transcription bubble. Mol Cell. 2015;59:258–269. 26186291 10.1016/j.molcel.2015.06.034 PMC4643057

[38] Nagano H, Fukudome A, Hiraguri A, et al. Distinct substrate specificities of Arabidopsis DCL3 and DCL4. Nucleic Acids Res. 2014;42:1845–1856. 24214956 10.1093/nar/gkt1077 PMC3919572

[39] Zilberman D, Cao X, Jacobsen SE. ARGONAUTE4 control of locus-specific siRNA accumulation and DNA and histone methylation. Science. 2003;299:716–719. 80-. 12522258 10.1126/science.1079695

[40] Duan C, Zhang H, Tang K, et al. Specific but interdependent functions for A rabidopsis AGO 4 and AGO 6 in RNA ‐directed DNA methylation. Embo J. 2015;34:581–592. 25527293 10.15252/embj.201489453 PMC4365029

[41] Havecker ER, Wallbridge LM, Hardcastle TJ, et al. The Arabidopsis RNA-directed DNA methylation argonautes functionally diverge based on their expression and interaction with target loci. Plant Cell. 2010;22:321–334. 20173091 10.1105/tpc.109.072199 PMC2845420

[42] McCue AD, Panda K, Nuthikattu S, et al. ARGONAUTE 6 bridges transposable element mRNA-derived siRNAs to the establishment of DNA methylation. Embo J. 2015;34:20–35. 25388951 10.15252/embj.201489499 PMC4291478

[43] Mi S, Cai T, Hu Y, et al. Sorting of small RNAs into Arabidopsis Argonaute complexes is directed by the 5' terminal nucleotide. Cell. 2008;133:116–127. 18342361 10.1016/j.cell.2008.02.034 PMC2981139

[44] Liu W, Duttke SH, Hetzel J, et al. RNA-directed DNA methylation involves co-transcriptional small-RNA-guided slicing of polymerase V transcripts in Arabidopsis. Nat Plants. 2018;4:181–188. 29379150 10.1038/s41477-017-0100-y PMC5832601

[45] Böhmdorfer G, Sethuraman S, Rowley MJ, et al. Long non-coding RNA produced by RNA polymerase V determines boundaries of heterochromatin. Elife. 2016;5. DOI:10.7554/eLife.19092. PMC5079748 27779094

[46] Marasco M, Li W, Lynch M, et al. Catalytic properties of RNA polymerases IV and V: accuracy, nucleotide incorporation and rNTP/dNTP discrimination. Nucleic Acids Res. 2017;45:11315–11326. 28977461 10.1093/nar/gkx794 PMC5737373

[47] Wierzbicki AT, Ream TS, Haag JR, et al. RNA polymerase V transcription guides ARGONAUTE4 to chromatin. Nat Genet. 2009;41:630–634. 19377477 10.1038/ng.365 PMC2674513

[48] El-Shami M, Pontier D, Lahmy S, et al. Reiterated WG/GW motifs form functionally and evolutionarily conserved ARGONAUTE-binding platforms in RNAi-related components. Genes Dev. 2007;21:2539–2544. 10.1101/gad.451207 PMC2000319 17938239

[49] Bies‐Etheve N, Pontier D, Lahmy S, et al. RNA‐directed DNA methylation requires an AGO4‐interacting member of the SPT5 elongation factor family. EMBO Rep. 2009;10:649–654. 19343051 10.1038/embor.2009.31 PMC2711833

[50] Lahmy S, Pontier D, Bies-Etheve N, et al. Evidence for ARGONAUTE4–DNA interactions in RNA-directed DNA methylation in plants. Genes Dev. 2016;30:2565–2570. 27986858 10.1101/gad.289553.116 PMC5204349

[51] Rowley MJ, Avrutsky MI, Sifuentes CJ, et al. Independent chromatin binding of ARGONAUTE4 and SPT5L/KTF1 mediates transcriptional gene silencing. PLoS Genet. 2011;7:e1002120. 21738482 10.1371/journal.pgen.1002120 PMC3111484

[52] Qi Y, He X, Wang XJ, et al. Distinct catalytic and non-catalytic roles of ARGONAUTE4 in RNA-directed DNA methylation. Nature. 2006;443:1008–1012. 16998468 10.1038/nature05198

[53] Ausin I, Greenberg MVC, Simanshu DK, et al. INVOLVED IN DE NOVO 2-containing complex involved in RNA-directed DNA methylation in Arabidopsis. Proc Natl Acad Sci. 2012;109:8374–8381. 22592791 10.1073/pnas.1206638109 PMC3365198

[54] Xie M, Ren G, Costa-Nunes P, et al. A subgroup of SGS3-like proteins act redundantly in RNA-directed DNA methylation. Nucleic Acids Res. 2012;40:4422–4431. 22302148 10.1093/nar/gks034 PMC3378875

[55] Zhang C-J, Ning Y-Q, Zhang S-W, et al. IDN2 and its paralogs form a complex required for RNA–directed DNA methylation. PLoS Genet. 2012;8:e1002693. 22570638 10.1371/journal.pgen.1002693 PMC3342958

[56] Law JA, Du J, Hale CJ, et al. Polymerase IV occupancy at RNA-directed DNA methylation sites requires SHH1. Nature. 2013;498:385–389. 23636332 10.1038/nature12178 PMC4119789

[57] Johnson LM, Du J, Hale CJ, et al. SRA- and SET-domain-containing proteins link RNA polymerase V occupancy to DNA methylation. Nature. 2014;507:124–128. 24463519 10.1038/nature12931 PMC3963826

[58] Mosher RA, Schwach F, Studholme D, et al. PolIVb influences RNA-directed DNA methylation independently of its role in siRNA biogenesis. Proc Natl Acad Sci U S A. 2008;105:3145–3150. 18287047 10.1073/pnas.0709632105 PMC2268599

[59] Wang F, Axtell MJ. AGO4 is specifically required for heterochromatic siRNA accumulation at Pol V-dependent loci in Arabidopsis thaliana. Plant J. 2017;90:37–47. 28002617 10.1111/tpj.13463

[60] Gallego-Bartolomé J, Liu W, Kuo PH, et al. Co-targeting RNA Polymerases IV and V promotes efficient de Novo DNA methylation in Arabidopsis. Cell. 2019;176:1068–1082.e19. 30739798 10.1016/j.cell.2019.01.029 PMC6386582

[61] Li Q, Gent JI, Zynda G, et al. RNA-directed DNA methylation enforces boundaries between heterochromatin and euchromatin in the maize genome. Proc Natl Acad Sci. 2015;112:14728–14733. 26553984 10.1073/pnas.1514680112 PMC4664327

[62] Haag JR, Brower-Toland B, Krieger EK, et al. Functional diversification of maize RNA Polymerase IV and V subtypes via alternative catalytic subunits. Cell Rep. 2014;9:378–390. 25284785 10.1016/j.celrep.2014.08.067 PMC4196699

[63] Smith LM, Pontes O, Searle I, et al. An SNF2 protein associated with nuclear RNA silencing and the spread of a silencing signal between cells in Arabidopsis. Plant Cell. 2007;19:1507–1521. 17526749 10.1105/tpc.107.051540 PMC1913737

[64] Hale CJ, Stonaker JL, Gross SM, et al. A novel Snf2 protein maintains trans-generational regulatory states established by paramutation in maize. PLoS Biol. 2007;5:e275. 17941719 10.1371/journal.pbio.0050275 PMC2020503

[65] Yang D-L, Zhang G, Wang L, et al. Four putative SWI2/SNF2 chromatin remodelers have dual roles in regulating DNA methylation in Arabidopsis. Cell Discov. 2018;4:55. 30345072 10.1038/s41421-018-0056-8 PMC6189096

[66] Zhou M, Palanca AMS, Law JA. Locus-specific control of the de novo DNA methylation pathway in Arabidopsis by the CLASSY family. Nat Genet. 2018;50:865–873. 29736015 10.1038/s41588-018-0115-y PMC6317521

[67] Zhang H, Ma Z-Y, Zeng L, et al. DTF1 is a core component of RNA-directed DNA methylation and may assist in the recruitment of Pol IV. Proc Natl Acad Sci. 2013;110:8290–8295. 23637343 10.1073/pnas.1300585110 PMC3657815

[68] Liu Z-W, Shao C-R, Zhang C-J, et al. The SET domain proteins SUVH2 and SUVH9 are required for Pol V occupancy at RNA-directed DNA methylation Loci. PLoS Genet. 2014;10:e1003948. 24465213 10.1371/journal.pgen.1003948 PMC3898904

[69] Liu Z-W, Zhou J-X, Huang H-W, et al. Two components of the RNA-directed DNA methylation pathway associate with MORC6 and silence Loci targeted by MORC6 in Arabidopsis. PLOS Genet. 2016;12:e1006026. 27171427 10.1371/journal.pgen.1006026 PMC4865133

[70] Jing Y, Sun H, Yuan W, et al. SUVH2 and SUVH9 couple two essential steps for transcriptional gene silencing in Arabidopsis. Mol Plant. 2016;9:1156–1167. 10.1016/j.molp.2016.05.006 27216319

[71] Moissiard G, Cokus SJ, Cary J, et al. MORC family ATPases required for heterochromatin condensation and gene silencing. Science. 2012;336:1448–1451. 22555433 10.1126/science.1221472 PMC3376212

[72] Law JA, Ausin I, Johnson LM, et al. A protein complex required for polymerase V transcripts and RNA- directed DNA methylation in Arabidopsis. Curr Biol. 2010;20:951–956. 20409711 10.1016/j.cub.2010.03.062 PMC2972704

[73] Zhong X, Hale CJ, Law JA, et al. DDR complex facilitates global association of RNA polymerase V to promoters and evolutionarily young transposons. Nat Struct Mol Biol. 2012;19:870–875. 22864289 10.1038/nsmb.2354 PMC3443314

[74] Wongpalee SP, Liu S, Gallego-Bartolomé J, et al. CryoEM structures of Arabidopsis DDR complexes involved in RNA-directed DNA methylation. Nat Commun. 2019;10:3916. 31477705 10.1038/s41467-019-11759-9 PMC6718625

[75] Gao Z, Liu H-L, Daxinger L, et al. An RNA polymerase II- and AGO4-associated protein acts in RNA-directed DNA methylation. Nature. 2010;465:106–109. 20410883 10.1038/nature09025 PMC2865564

[76] Allard STM, Bingman CA, Johnson KA, et al. Structure at 1.6 Å resolution of the protein from gene locus At3g22680 from Arabidopsis thaliana. Acta Crystallogr Sect F Struct Biol Cryst Commun. 2005;61:647–650. 10.1107/S1744309105019743 PMC1952470 16511118

[77] Kanno T, Bucher E, Daxinger L, et al. A structural-maintenance-of-chromosomes hinge domain–containing protein is required for RNA-directed DNA methylation. Nat Genet. 2008;40:670–675. 18425128 10.1038/ng.119

[78] Kanno T, Mette MF, Kreil DP, et al. Involvement of putative SNF2 Chromatin remodeling protein DRD1 in RNA-directed DNA methylation. Curr Biol. 2004;14:801–805. 15120073 10.1016/j.cub.2004.04.037

[79] Cramer P. Structural basis of transcription: RNA polymerase II at 2.8 Angstrom resolution. Science. 2001;292:1863–1876. 11313498 10.1126/science.1059493

[80] Cramer P. Architecture of RNA polymerase II and implications for the transcription mechanism. Science. 2000;288:640–649. 10784442 10.1126/science.288.5466.640

[81] Armache K-J, Mitterweger S, Meinhart A, et al. Structures of complete RNA polymerase II and its subcomplex, Rpb4/7. J Biol Chem. 2005;280:7131–7134. 15591044 10.1074/jbc.M413038200

[82] Fernández-Tornero C, Moreno-Morcillo M, Rashid UJ, et al. Crystal structure of the 14-subunit RNA polymerase I. Nature. 2013;502:644–649. 24153184 10.1038/nature12636

[83] Engel C, Sainsbury S, Cheung AC, et al. RNA polymerase I structure and transcription regulation. Nature. 2013;502:650–655. 24153182 10.1038/nature12712

[84] Hoffmann NA, Jakobi AJ, Moreno-Morcillo M, et al. Molecular structures of unbound and transcribing RNA polymerase III. Nature. 2015;528:231–236. 26605533 10.1038/nature16143 PMC4681132

[85] Cramer P. Organization and regulation of gene transcription. Nature. 2019;573:45–54. 31462772 10.1038/s41586-019-1517-4

[86] Werner F, Grohmann D. Evolution of multisubunit RNA polymerases in the three domains of life. Nat Rev Microbiol. 2011;9:85–98. 21233849 10.1038/nrmicro2507

[87] Fouqueau T, Blombach F, Werner F. Evolutionary origins of two-barrel RNA polymerases and site-specific transcription initiation. Annu Rev Microbiol. 2017;71:331–348. 28657884 10.1146/annurev-micro-091014-104145

[88] Chakraborty A, Wang D, Ebright YW, et al. Opening and closing of the bacterial RNA polymerase clamp. Science. 2012;337:591–595. 22859489 10.1126/science.1218716 PMC3626110

[89] Khatter H, Vorländer MK, Müller CW. RNA polymerase I and III: similar yet unique. Curr Opin Struct Biol. 2017;47:88–94. 28743025 10.1016/j.sbi.2017.05.008

[90] Sosunov V. Unified two-metal mechanism of RNA synthesis and degradation by RNA polymerase. Embo J. 2003;22:2234–2244. 12727889 10.1093/emboj/cdg193 PMC156065

[91] Minakhin L, Bhagat S, Brunning A, et al. Bacterial RNA polymerase subunit and eukaryotic RNA polymerase subunit RPB6 are sequence, structural, and functional homologs and promote RNA polymerase assembly. Proc Natl Acad Sci. 2001;98:892–897. 11158566 10.1073/pnas.98.3.892 PMC14680

[92] Li Y, Yuan Y, Fang X, et al. A Role for MINIYO and QUATRE-QUART2 in the assembly of RNA polymerases II, IV, and V in Arabidopsis. Plant Cell. 2018;30:466–480. 29352065 10.1105/tpc.17.00380 PMC5868690

[93] Allison LA, Moyle M, Shales M, et al. Extensive homology among the largest subunits of eukaryotic and prokaryotic RNA polymerases. Cell. 1985;42:599–610. 3896517 10.1016/0092-8674(85)90117-5

[94] Sweetser D, Nonet M, Young RA. Prokaryotic and eukaryotic RNA polymerases have homologous core subunits. Proc Natl Acad Sci. 1987;84:1192–1196. 3547406 10.1073/pnas.84.5.1192 PMC304392

[95] Zaborowska J, Egloff S, Murphy S. The pol II CTD: new twists in the tail. Nat Struct Mol Biol. 2016;23:771–777. 27605205 10.1038/nsmb.3285

[96] Koiwa H, Hausmann S, Bang WY, et al. Arabidopsis C-terminal domain phosphatase-like 1 and 2 are essential Ser-5-specific C-terminal domain phosphatases. Proc Natl Acad Sci. 2004;101:14539–14544. 15388846 10.1073/pnas.0403174101 PMC521950

[97] Trujillo JT, Beilstein MA, Mosher RA. The Argonaute-binding platform of NRPE1 evolves through modulation of intrinsically disordered repeats. New Phytol. 2016;212:1094–1105. 27431917 10.1111/nph.14089 PMC5125548

[98] Pontier D, Picart C, Roudier F, et al. NERD, a plant-specific GW protein, defines an additional RNAi-dependent Chromatin-based pathway in Arabidopsis. Mol Cell. 2012;48:121–132. 22940247 10.1016/j.molcel.2012.07.027

[99] Wendte JM, Haag JR, Singh J, et al. Functional dissection of the Pol V largest subunit CTD in RNA-directed DNA methylation. Cell Rep. 2017;19:2796–2808. 28658626 10.1016/j.celrep.2017.05.091 PMC5541899

[100] Zhang H, Tang K, Qian W, et al. An Rrp6-like protein positively regulates noncoding RNA levels and DNA Methylation in Arabidopsis. Mol Cell. 2014;54:418–430. 24726328 10.1016/j.molcel.2014.03.019 PMC4023806

[101] Wendte JM, Haag JR, Pontes OM, et al. The Pol IV largest subunit CTD quantitatively affects siRNA levels guiding RNA-directed DNA methylation. Nucleic Acids Res. 2019;47:9024–9036. 31329950 10.1093/nar/gkz615 PMC6753486

[102] Ferrafiat L, Pflieger D, Singh J, et al. The NRPD1 N-terminus contains a Pol IV-specific motif that is critical for genome surveillance in Arabidopsis. Nucleic Acids Res. 2019;47:9037–9052. 31372633 10.1093/nar/gkz618 PMC6753494

[103] Huang X, Wang D, Weiss DR, et al. RNA polymerase II trigger loop residues stabilize and position the incoming nucleotide triphosphate in transcription. Proc Natl Acad Sci. 2010;107:15745–15750. 20798057 10.1073/pnas.1009898107 PMC2936645

[104] Erhard KF, Stonaker JL, Parkinson SE, et al. RNA Polymerase IV functions in paramutation in Zea mays. Science. 2009;323:1201–1205. 19251626 10.1126/science.1164508

[105] Kaplan CD, Larsson K-M, Kornberg RD. The RNA polymerase II trigger loop functions in substrate selection and is directly targeted by alpha-amanitin. Mol Cell. 2008;30:547–556. 18538653 10.1016/j.molcel.2008.04.023 PMC2475549

[106] Toulokhonov I, Zhang J, Palangat M, et al. A central role of the RNA polymerase trigger loop in active-site rearrangement during transcriptional pausing. Mol Cell. 2007;27:406–419. 17679091 10.1016/j.molcel.2007.06.008

[107] Huang Y, Kendall T, Forsythe ES, et al. Ancient origin and recent innovations of RNA Polymerase IV and V. Mol Biol Evol. 2015;32:1788–1799. 25767205 10.1093/molbev/msv060 PMC4476159

[108] Tucker SL, Reece J, Ream TS, et al. Evolutionary history of plant multisubunit RNA polymerases IV and V: subunit origins via genome-wide and segmental gene duplications, retrotransposition, and lineage-specific subfunctionalization. Cold Spring Harb Symp Quant Biol. 2010;75:285–297. 21447813 10.1101/sqb.2010.75.037

[109] Aguilar‐Cruz A, Grimanelli D, Haseloff J, et al. DNA methylation in Marchantia polymorpha. New Phytol. 2019;223:575–581. 30920664 10.1111/nph.15818

[110] Coruh C, Cho SH, Shahid S, et al. Comprehensive annotation of physcomitrella patens small RNA Loci reveals that the heterochromatic short interfering RNA pathway is largely conserved in land plants. Plant Cell. 2015;27:2148–2162. 26209555 10.1105/tpc.15.00228 PMC4568501

[111] Gouil Q, Baulcombe DC, Mittelsten Scheid O. DNA methylation signatures of the plant chromomethyltransferases. PLOS Genet. 2016;12:e1006526. 27997534 10.1371/journal.pgen.1006526 PMC5221884

[112] Xu L, Yuan K, Yuan M, et al. Regulation of rice tillering by RNA-directed DNA methylation at miniature inverted-repeat transposable elements. Mol Plant. 2020;13:851–863. 10.1016/j.molp.2020.02.009 32087371

[113] Wei L, Gu L, Song X, et al. Dicer-like 3 produces transposable element-associated 24-nt siRNAs that control agricultural traits in rice. Proc Natl Acad Sci. 2014;111:3877–3882. 24554078 10.1073/pnas.1318131111 PMC3956178

[114] Tan F, Zhou C, Zhou Q, et al. Analysis of Chromatin regulators reveals specific features of rice DNA methylation pathways. Plant Physiol. 2016;171:2041–2054. 27208249 10.1104/pp.16.00393 PMC4936571

[115] Sidorenko L, Dorweiler JE, Cigan AM, et al. A dominant mutation in mediator of paramutation2, one of three second-largest subunits of a plant-specific RNA polymerase, disrupts multiple siRNA silencing processes. PLoS Genet. 2009;5:e1000725. 19936058 10.1371/journal.pgen.1000725 PMC2774164

[116] Stonaker JL, Lim JP, Erhard KF, et al. Diversity of Pol IV function is defined by mutations at the Maize rmr7 Locus. PLoS Genet. 2009;5:e1000706. 19936246 10.1371/journal.pgen.1000706 PMC2775721

[117] Alleman M, Sidorenko L, McGinnis K, et al. An RNA-dependent RNA polymerase is required for paramutation in maize. Nature. 2006;442:295–298. 16855589 10.1038/nature04884

[118] Grover JW, Kendall T, Baten A, et al. Maternal components of RNA‐directed DNA methylation are required for seed development in Brassica rapa. Plant J. 2018;94:575–582. 29569777 10.1111/tpj.13910

[119] Wang Z, Butel N, Santos-González J, et al. Polymerase IV plays a crucial role in pollen development in Capsella. Plant Cell. 2020;32:950–966. 31988265 10.1105/tpc.19.00938 PMC7145478

[120] Parkinson SE, Gross SM, Hollick JB. Maize sex determination and abaxial leaf fates are canalized by a factor that maintains repressed epigenetic states. Dev Biol. 2007;308:462–473. 17612519 10.1016/j.ydbio.2007.06.004

[121] Dorweiler JE, Carey CC, Kubo KM, et al. mediator of paramutation1 is required for establishment and maintenance of paramutation at multiple Maize Loci. Plant Cell. 2000;12:2101–2118. 11090212 10.1105/tpc.12.11.2101 PMC150161

[122] Popova OV, Dinh HQ, Aufsatz W, et al. The RdDM pathway is required for Basal heat tolerance in Arabidopsis. Mol Plant. 2013;6:396–410. 23376771 10.1093/mp/sst023 PMC3603006

[123] Schalk C, Cognat V, Graindorge S, et al. Small RNA-mediated repair of UV-induced DNA lesions by the DNA Damage-Binding Protein 2 and Argonaute 1. Proc Natl Acad Sci. 2017;114:E2965–E2974. 28325872 10.1073/pnas.1618834114 PMC5389294

[124] Mao H, Wang H, Liu S, et al. A transposable element in a NAC gene is associated with drought tolerance in maize seedlings. Nat Commun. 2015;6:8326. 26387805 10.1038/ncomms9326 PMC4595727

[125] Dowen RH, Pelizzola M, Schmitz RJ, et al. Widespread dynamic DNA methylation in response to biotic stress. Proc Natl Acad Sci. 2012;109:E2183–E2191. 22733782 10.1073/pnas.1209329109 PMC3420206

[126] López A, Ramírez V, García-Andrade J, et al. The RNA silencing enzyme RNA Polymerase V is required for plant immunity. PLoS Genet. 2011;7:e1002434. 22242006 10.1371/journal.pgen.1002434 PMC3248562

[127] Yu A, Lepere G, Jay F, et al. Dynamics and biological relevance of DNA demethylation in Arabidopsis antibacterial defense. Proc Natl Acad Sci. 2013;110:2389–2394. 23335630 10.1073/pnas.1211757110 PMC3568381

[128] Le T-N, Schumann U, Smith NA, et al. DNA demethylases target promoter transposable elements to positively regulate stress responsive genes in Arabidopsis. Genome Biol. 2014;15:458. 25228471 10.1186/s13059-014-0458-3 PMC4189188

[129] Zhang H, Tao Z, Hong H, et al. Transposon-derived small RNA is responsible for modified function of WRKY45 locus. Nat Plants. 2016;2:16016. 27249351 10.1038/nplants.2016.16

[130] Chow HT, Chakraborty T, Mosher RA. RNA-directed DNA methylation and sexual reproduction: expanding beyond the seed. Curr Opin Plant Biol. 2019;54:11–17. 31881293 10.1016/j.pbi.2019.11.006

[131] Kinoshita Y, Saze H, Kinoshita T, et al. Control of FWA gene silencing in Arabidopsis thaliana by SINE-related direct repeats. Plant J. 2006;49:38–45. 17144899 10.1111/j.1365-313X.2006.02936.x

[132] Ito H, Gaubert H, Bucher E, et al. An siRNA pathway prevents transgenerational retrotransposition in plants subjected to stress. Nature. 2011;472:115–119. 21399627 10.1038/nature09861

[133] Thieme M, Lanciano S, Balzergue S, et al. Inhibition of RNA polymerase II allows controlled mobilisation of retrotransposons for plant breeding. Genome Biol. 2017;18:134. 28687080 10.1186/s13059-017-1265-4 PMC5501947

[134] Pietzenuk B, Markus C, Gaubert H, et al. Recurrent evolution of heat-responsiveness in Brassicaceae COPIA elements. Genome Biol. 2016;17:209. 27729060 10.1186/s13059-016-1072-3 PMC5059998

[135] Cavrak VV, Lettner N, Jamge S, et al. How a retrotransposon exploits the plant’s heat stress response for its activation. PLoS Genet. 2014;10:e1004115. 24497839 10.1371/journal.pgen.1004115 PMC3907296

[136] Benoit M, Drost H-G, Catoni M, et al. Environmental and epigenetic regulation of rider retrotransposons in tomato. PLoS Genet. 2019;15:e1008370. 31525177 10.1371/journal.pgen.1008370 PMC6762207

[137] Mirouze M, Reinders J, Bucher E, et al. Selective epigenetic control of retrotransposition in Arabidopsis. Nature. 2009;461:427–430. 19734882 10.1038/nature08328

[138] Marí-Ordóñez A, Marchais A, Etcheverry M, et al. Reconstructing de novo silencing of an active plant retrotransposon. Nat Genet. 2013;45:1029–1039. 23852169 10.1038/ng.2703

[139] Quadrana L, Etcheverry M, Gilly A, et al. Transposition favors the generation of large effect mutations that may facilitate rapid adaption. Nat Commun. 2019;10:3421. 31366887 10.1038/s41467-019-11385-5 PMC6668482

[140] Tsukahara S, Kobayashi A, Kawabe A, et al. Bursts of retrotransposition reproduced in Arabidopsis. Nature. 2009;461:423–426. 19734880 10.1038/nature08351

[141] Lippman Z, Gendrel A-V, Black M, et al. Role of transposable elements in heterochromatin and epigenetic control. Nature. 2004;430:471–476. 15269773 10.1038/nature02651

[142] Henderson IR, Jacobsen SE. Tandem repeats upstream of the Arabidopsis endogene SDC recruit non-CG DNA methylation and initiate siRNA spreading. Genes Dev. 2008;22:1597–1606. 18559476 10.1101/gad.1667808 PMC2428058

[143] Wang P-H, Wittmeyer KT, Lee T-F, et al. Overlapping RdDM and non-RdDM mechanisms work together to maintain somatic repression of a paramutagenic epiallele of maize pericarp color1. PLoS One. 2017;12:e0187157. 29112965 10.1371/journal.pone.0187157 PMC5675401

[144] Fujimoto R, Kinoshita Y, Kawabe A, et al. Evolution and control of imprinted FWA genes in the genus Arabidopsis. PLoS Genet. 2008;4:e1000048. 18389059 10.1371/journal.pgen.1000048 PMC2270340

[145] Chan SWL, Zilberman D, Xie Z, et al. RNA silencing genes control de novo DNA methylation. Science. 2004;80(–303):1336. 10.1126/science.1095989 14988555

[146] Soppe WJJ, Jacobsen SE, Alonso-Blanco C, et al. The late flowering phenotype of fwa mutants is caused by gain-of-function epigenetic alleles of a homeodomain gene. Mol Cell. 2000;6:791–802. 11090618 10.1016/s1097-2765(05)00090-0

[147] Jiao Y, Wang Y, Xue D, et al. Regulation of OsSPL14 by OsmiR156 defines ideal plant architecture in rice. Nat Genet. 2010;42:541–544. 20495565 10.1038/ng.591

[148] Wassenegger M, Heimes S, Riedel L, et al. RNA-directed de novo methylation of genomic sequences in plants. Cell. 1994;76:567–576. 8313476 10.1016/0092-8674(94)90119-8

[149] Mette MF, Aufsatz W, van der Winden J, et al. Transcriptional silencing and promoter methylation triggered by double-stranded RNA. Embo J. 2000;19:5194–5201. 11013221 10.1093/emboj/19.19.5194 PMC302106

[150] Jones L, Hamilton AJ, Voinnet O, et al. RNA-DNA interactions and DNA methylation in post-transcriptional gene silencing. Plant Cell. 1999;11:2291–2301. 10590159 10.1105/tpc.11.12.2291 PMC144133

[151] Hollick JB. Paramutation and related phenomena in diverse species. Nat Rev Genet. 2017;18:5–23. 27748375 10.1038/nrg.2016.115

[152] Brink RA. A genetic change associated with the R Locus in Maize which is directed and potentially reversible. Genetics. 1956;41:872–889. 17247669 10.1093/genetics/41.6.872 PMC1224369

[153] Sloan AE, Sidorenko L, McGinnis KM. Diverse gene-silencing mechanisms with distinct requirements for RNA polymerase subunits in Zea mays. Genetics. 2014;198(3):1031–1042. 25164883 10.1534/genetics.114.168518 PMC4224150

[154] Hale CJ, Erhard KF, Lisch D, et al. Production and processing of siRNA precursor transcripts from the highly repetitive Maize genome. PLoS Genet. 2009;5:e1000598. 19680464 10.1371/journal.pgen.1000598 PMC2725412

[155] Chandler VL. Paramutation: from Maize to Mice. Cell. 2007;128:641–645. 17320501 10.1016/j.cell.2007.02.007

[156] Gouil Q, Baulcombe DC. Paramutation-like features of multiple natural epialleles in tomato. BMC Genomics. 2018;19:203. 29554868 10.1186/s12864-018-4590-4 PMC5859443

[157] Ma Z, Coruh C, Axtell MJ. Arabidopsis lyrata small RNAs: transient MIRNA and small interfering RNA loci within the Arabidopsis genus. Plant Cell. 2010;22:1090–1103. 20407023 10.1105/tpc.110.073882 PMC2879747

[158] Lunardon A, Johnson NR, Hagerott E, et al. Integrated annotations and analyses of small RNA–producing loci from 47 diverse plants. Genome Res. 2020;30:497–513. 32179590 10.1101/gr.256750.119 PMC7111516

[159] Axtell MJ, Bartel DP. Antiquity of microRNAs and their targets in land plants. Plant Cell. 2005;17:1658–1673. 15849273 10.1105/tpc.105.032185 PMC1143068

[160] Naydenov M, Baev V, Apostolova E, et al. High-temperature effect on genes engaged in DNA methylation and affected by DNA methylation in Arabidopsis. Plant Physiol Biochem. 2015;87:102–108. 25576840 10.1016/j.plaphy.2014.12.022

[161] Bouyer D, Kramdi A, Kassam M, et al. DNA methylation dynamics during early plant life. Genome Biol. 2017;18. DOI:10.1186/s13059-017-1313-0. PMC5611644 28942733

[162] Walker J, Gao H, Zhang J, et al. Sexual-lineage-specific DNA methylation regulates meiosis in Arabidopsis. Nat Genet. 2018;50:130–137. 29255257 10.1038/s41588-017-0008-5 PMC7611288

[163] Narsai R, Gouil Q, Secco D, et al. Extensive transcriptomic and epigenomic remodelling occurs during Arabidopsis thaliana germination. Genome Biol. 2017;18:172. 28911330 10.1186/s13059-017-1302-3 PMC5599894

[164] Kawakatsu T, Nery JR, Castanon R, et al. Dynamic DNA methylation reconfiguration during seed development and germination. Genome Biol. 2017;18:171. 28911331 10.1186/s13059-017-1251-x PMC5599895

[165] Gehring M. Epigenetic dynamics during flowering plant reproduction: evidence for reprogramming? New Phytol. 2019;224:91–96. 31002174 10.1111/nph.15856 PMC6711810

[166] Batista RA, Köhler C. Genomic imprinting in plants—revisiting existing models. Genes Dev. 2020;34:24–36. 31896690 10.1101/gad.332924.119 PMC6938664

[167] Baubec T, Finke A, Mittelsten Scheid O, et al. Meristem-specific expression of epigenetic regulators safeguards transposon silencing in Arabidopsis. EMBO Rep. 2014;15:446–452. 10.1002/embr.201337915 PMC3989676 24562611

[168] Zhang C, Wei Y, Xu L, et al. A Bunyavirus-inducible Ubiquitin Ligase targets RNA Polymerase IV for degradation during viral pathogenesis in rice. Mol Plant. 2020. DOI:10.1016/j.molp.2020.02.010. 32087369

[169] Zhong S, Xu Y, Yu C, et al. Anaphase-promoting complex/cyclosome regulates RdDM activity by degrading DMS3 in Arabidopsis. Proc Natl Acad Sci. 2019;116:3899–3908. 30760603 10.1073/pnas.1816652116 PMC6397581

[170] Melnyk CW, Molnar A, Bassett A, et al. Mobile 24 nt Small RNAs Direct Transcriptional Gene Silencing in the Root Meristems of Arabidopsis thaliana. Curr Biol. 2011;21:1678–1683. 21962713 10.1016/j.cub.2011.08.065

[171] Molnar A, Melnyk CW, Bassett A, et al. Small silencing RNAs in plants are mobile and direct epigenetic modification in recipient cells. Science. 2010;328:872–875. (80-). 20413459 10.1126/science.1187959

[172] Olmedo-Monfil V, Durán-Figueroa N, Arteaga-Vázquez M, et al. Control of female gamete formation by a small RNA pathway in Arabidopsis. Nature. 2010;464:628–632. 20208518 10.1038/nature08828 PMC4613780

[173] Martínez G, Panda K, Köhler C, et al. Silencing in sperm cells is directed by RNA movement from the surrounding nurse cell. Nat Plants. 2016;2:16030. 27249563 10.1038/nplants.2016.30

[174] Slotkin RK, Vaughn M, Borges F, et al. Epigenetic reprogramming and small RNA silencing of transposable elements in Pollen. Cell. 2009;136:461–472. 19203581 10.1016/j.cell.2008.12.038 PMC2661848

[175] Lewsey MG, Hardcastle TJ, Melnyk CW, et al. Mobile small RNAs regulate genome-wide DNA methylation. Proc Natl Acad Sci. 2016;113:E801–E810. 26787884 10.1073/pnas.1515072113 PMC4760824

[176] Li Q, Eichten SR, Hermanson PJ, et al. Genetic perturbation of the Maize Methylome. Plant Cell. 2014;26:4602–4616. 25527708 10.1105/tpc.114.133140 PMC4311211

[177] Blevins T, Pontvianne F, Cocklin R, et al. A two-step process for epigenetic inheritance in Arabidopsis. Mol Cell. 2014;54:30–42. 24657166 10.1016/j.molcel.2014.02.019 PMC3988221

[178] Deleris A, Greenberg MVC, Ausin I, et al. Involvement of a Jumonji‐C domain‐containing histone demethylase in DRM2‐mediated maintenance of DNA methylation. EMBO Rep. 2010;11:950–955. 21052090 10.1038/embor.2010.158 PMC2999860

[179] Searle IR, Pontes O, Melnyk CW, et al. JMJ14, a JmjC domain protein, is required for RNA silencing and cell-to-cell movement of an RNA silencing signal in Arabidopsis. Genes Dev. 2010;24:986–991. 20478993 10.1101/gad.579910 PMC2867213

[180] Mathieu O, Reinders J, Caikovski M, et al. Transgenerational stability of the Arabidopsis epigenome is coordinated by CG methylation. Cell. 2007;130:851–862. 17803908 10.1016/j.cell.2007.07.007

[181] Blevins T, Pontes O, Pikaard CS, et al. Heterochromatic siRNAs and DDM1 independently silence aberrant 5S rDNA transcripts in Arabidopsis. PLoS One. 2009;4:e5932. 19529764 10.1371/journal.pone.0005932 PMC2691480

[182] Earley KW, Pontvianne F, Wierzbicki AT, et al. Mechanisms of HDA6-mediated rRNA gene silencing: suppression of intergenic Pol II transcription and differential effects on maintenance versus siRNA-directed cytosine methylation. Genes Dev. 2010;24:1119–1132. 20516197 10.1101/gad.1914110 PMC2878650

[183] Pecinka A, Dinh HQ, Baubec T, et al. Epigenetic regulation of repetitive elements is attenuated by prolonged heat stress in Arabidopsis. Plant Cell. 2010;22:3118–3129. 20876829 10.1105/tpc.110.078493 PMC2965555

[184] Teixeira FK, Heredia F, Sarazin A, et al. A role for RNAi in the selective correction of DNA methylation defects. Science. 2009;323:1600–1604. (80-). 19179494 10.1126/science.1165313

[185] Gong Z, Morales-Ruiz T, Ariza RR, et al. ROS1, a repressor of transcriptional gene silencing in Arabidopsis, encodes a DNA Glycosylase/Lyase. Cell. 2002;111:803–814. 12526807 10.1016/s0092-8674(02)01133-9

[186] Penterman J, Zilberman D, Huh JH, et al. DNA demethylation in the Arabidopsis genome. Proc Natl Acad Sci. 2007;104:6752–6757. 17409185 10.1073/pnas.0701861104 PMC1847597

[187] Lei M, Zhang H, Julian R, et al. Regulatory link between DNA methylation and active demethylation in Arabidopsis. Proc Natl Acad Sci. 2015;112:3553–3557. 25733903 10.1073/pnas.1502279112 PMC4371987

[188] Ibarra CA, Feng X, Schoft VK, et al. Active DNA demethylation in plant companion cells reinforces transposon methylation in Gametes. Science. 2012;337:1360–1364. (80-). 22984074 10.1126/science.1224839 PMC4034762

[189] Gehring M, Huh JH, Hsieh T-F, et al. DEMETER DNA glycosylase establishes MEDEA polycomb gene self-imprinting by Allele-specific demethylation. Cell. 2006;124:495–506. 16469697 10.1016/j.cell.2005.12.034 PMC4106368

[190] Hsieh TF, Ibarra CA, Silva P, et al. Genome-wide demethylation of Arabidopsis endosperm. Science. 2009;324:1451–1454. (80-). 10.1126/science.1172417 PMC4044190 19520962

[191] Trujillo JT, Seetharam AS, Hufford MB, et al. Evidence for a unique DNA-dependent RNA polymerase in cereal crops. Mol Biol Evol. 2018;35:2454–2462. 30053133 10.1093/molbev/msy146 PMC6188566

[192] Wei W, Ba Z, Gao M, et al. A role for small RNAs in DNA double-strand break repair. Cell. 2012;149:101–112. 22445173 10.1016/j.cell.2012.03.002

[193] Graindorge S, Cognat V, Johann to Berens P, et al. Photodamage repair pathways contribute to the accurate maintenance of the DNA methylome landscape upon UV exposure. PLOS Genet. 2019;15:e1008476. 31738755 10.1371/journal.pgen.1008476 PMC6886878

[194] Nuthikattu S, McCue AD, Panda K, et al. The initiation of epigenetic silencing of active transposable elements is triggered by RDR6 and 21-22 nucleotide small interfering RNAs. Plant Physiol. 2013;162:116–131. 23542151 10.1104/pp.113.216481 PMC3641197

[195] Taochy C, Yu A, Bouché N, et al. Post-transcriptional gene silencing triggers dispensable DNA methylation in gene body in Arabidopsis. Nucleic Acids Res. 2019;47:9104–9114. 31372641 10.1093/nar/gkz636 PMC6753489

[196] Cuerda-Gil D, Slotkin RK. Non-canonical RNA-directed DNA methylation. Nat Plants. 2016;2:16163. 27808230 10.1038/nplants.2016.163

[197] Vazquez F, Blevins T, Ailhas J, et al. Evolution of Arabidopsis MIR genes generates novel microRNA classes. Nucleic Acids Res. 2008;36:6429–6438. 18842626 10.1093/nar/gkn670 PMC2582634

[198] Panda K, Ji L, Neumann DA, et al. Full-length autonomous transposable elements are preferentially targeted by expression-dependent forms of RNA-directed DNA methylation. Genome Biol. 2016;17:170. 27506905 10.1186/s13059-016-1032-y PMC4977677

[199] Wu L, Zhou H, Zhang Q, et al. DNA methylation mediated by a microRNA pathway. Mol Cell. 2010;38:465–475. 20381393 10.1016/j.molcel.2010.03.008

[200] Chen D, Meng Y, Yuan C, et al. Plant siRNAs from introns mediate DNA methylation of host genes. RNA. 2011;17:1012–1024. 21518803 10.1261/rna.2589011 PMC3096033

[201] Ariel F, Jegu T, Latrasse D, et al. Noncoding transcription by alternative RNA polymerases dynamically regulates an auxin-driven chromatin loop. Mol Cell. 2014;55:383–396. 25018019 10.1016/j.molcel.2014.06.011

[202] Dalmay T, Hamilton A, Rudd S, et al. An RNA-dependent RNA polymerase gene in Arabidopsis is required for posttranscriptional gene silencing mediated by a transgene but not by a virus. Cell. 2000;101:543–553. 10850496 10.1016/s0092-8674(00)80864-8

[203] Mourrain P, Beclin C, Elmayan T, et al. Arabidopsis SGS2 and SGS3 genes are required for posttranscriptional gene silencing and natural virus resistance. Cell. 2000;101:533–542. 10850495 10.1016/s0092-8674(00)80863-6

[204] Talmor-Neiman M, Stav R, Klipcan L, et al. Identification of trans-acting siRNAs in moss and an RNA-dependent RNA polymerase required for their biogenesis. Plant J. 2006;48:511–521. 17076803 10.1111/j.1365-313X.2006.02895.x

[205] Schalk C, Drevensek S, Kramdi A, et al. DNA DAMAGE BINDING PROTEIN 2 (DDB2) Shapes the DNA Methylation Landscape. Plant Cell. 2016;28:2043–2059. 10.1105/tpc.16.00474 PMC5059809 27531226

[206] He XJ, Hsu YF, Pontes O, et al. NRPD4, a protein related to the RPB4 subunit of RNA polymerase II, is a component of RNA polymerases IV and V and is required for RNA-directed DNA methylation. Genes Dev. 2009;23:318–330. 19204117 10.1101/gad.1765209 PMC2648547

[207] Haag JR, Pontes O, Pikaard CS. Metal A and metal B sites of nuclear RNA polymerases Pol IV and Pol V are required for siRNA-dependent DNA methylation and gene silencing. PLoS One. 2009;4:e4110. 19119310 10.1371/journal.pone.0004110 PMC2605557

[208] Pires ND, Dolan L. Morphological evolution in land plants: new designs with old genes. Philos Trans R Soc B Biol Sci. 2012;367:508–518. 10.1098/rstb.2011.0252 PMC3248709 22232763

